# Genomic analyses identify recurrent *MEF2D* fusions in acute lymphoblastic leukaemia

**DOI:** 10.1038/ncomms13331

**Published:** 2016-11-08

**Authors:** Zhaohui Gu, Michelle Churchman, Kathryn Roberts, Yongjin Li, Yu Liu, Richard C. Harvey, Kelly McCastlain, Shalini C. Reshmi, Debbie Payne-Turner, Ilaria Iacobucci, Ying Shao, I-Ming Chen, Marcus Valentine, Deqing Pei, Karen L. Mungall, Andrew J. Mungall, Yussanne Ma, Richard Moore, Marco Marra, Eileen Stonerock, Julie M. Gastier-Foster, Meenakshi Devidas, Yunfeng Dai, Brent Wood, Michael Borowitz, Eric E. Larsen, Kelly Maloney, Leonard A. Mattano Jr, Anne Angiolillo, Wanda L. Salzer, Michael J. Burke, Francesca Gianni, Orietta Spinelli, Jerald P. Radich, Mark D. Minden, Anthony V. Moorman, Bella Patel, Adele K. Fielding, Jacob M. Rowe, Selina M. Luger, Ravi Bhatia, Ibrahim Aldoss, Stephen J. Forman, Jessica Kohlschmidt, Krzysztof Mrózek, Guido Marcucci, Clara D. Bloomfield, Wendy Stock, Steven Kornblau, Hagop M. Kantarjian, Marina Konopleva, Elisabeth Paietta, Cheryl L. Willman, Mignon L. Loh, Stephen P. Hunger, Charles G. Mullighan

**Affiliations:** 1 Department of Pathology and Hematological Malignancies Program, St Jude Children's Research Hospital, 262 Danny Thomas Place, MS 342, Memphis, Tennessee 38105, USA; 2 Department of Computational Biology, St Jude Children's Research Hospital, Memphis, Tennessee 38105, USA; 3 University of New Mexico Cancer Center, Albuquerque, New Mexico 87106, USA; 4 The Research Institute, Nationwide Children's Hospital, Columbus, Ohio 43205, USA; 5 Cytogenetic Shared Resource, St Jude Children's Research Hospital, Memphis, Tennessee 38105, USA; 6 Department of Biostatistics, St Jude Children's Research Hospital, Memphis, Tennessee 38105; 7 Canada's Michael Smith Genome Sciences Centre, BC Cancer Agency, Vancouver, BC V5Z 4S6, Canada; 8 Department of Pathology and Laboratory Medicine, Nationwide Children's Hospital, Columbus, Ohio 43205, USA; 9 Department of Pathology, The Ohio State University College of Medicine, Columbus, Ohio 43210, USA; 10 Department of Pediatrics, The Ohio State University College of Medicine, Columbus, Ohio 43210, USA; 11 Department of Biostatistics, Colleges of Medicine and Public Health & Health Professions, University of Florida, Gainesville, Florida 32611, USA; 12 Department of Laboratory Medicine, University of Washington, Seattle, Washington 98195, USA; 13 Department of Pathology, Johns Hopkins Medical Institutions, Baltimore, Maryland 21287, USA; 14 Maine Children's Cancer Program, Scarborough, Maine 04074, USA; 15 Pediatric Hematology/Oncology/BMT, University of Colorado School of Medicine and Children's Hospital Colorado, Aurora, Colorado 80045, USA; 16 HARP Pharma Consulting, Mystic, Connecticut 06355, USA; 17 Children's National Medical Center, Washington, DC 20010, USA; 18 US Army Medical Research and Materiel Command, Fort Detrick, Maryland 21702, USA; 19 Medical College of Wisconsin, Milwaukee, Wisconsin 53226, USA; 20 Department of Hematology and Bone Marrow Transplantation, Papa Giovanni XXIII Hospital Piazza OMS 1 24127, Bergamo, Italy; 21 Fred Hutchinson Cancer Research Center, Seattle, Washington 98109, USA; 22 Princess Margaret Cancer Centre, University Health Network, Toronto, ON M5G 2M9, Canada; 23 Leukemia Research Cytogenetics Group, Northern Institute for Cancer Research, Newcastle University, Newcastle upon Tyne NE1 7RU, UK; 24 Department of Haemato-Oncology, Barts Cancer Institute, London EC1M 6BQ, UK; 25 Department of Hematology, UCL Cancer Institute, London WC1E 6BT, UK; 26 Hematology, Shaare Zedek Medical Center, Jerusalem 9103102, Israel; 27 Abramson Cancer Center, University of Pennsylvania, Philadelphia, Pennsylvania 19104, USA; 28 Division of Hematology and Oncology, Department of Medicine, The University of Alabama at Birmingham, Birmingham, Alabama 35294, USA; 29 Gehr Family Center for Leukemia Research, City of Hope, Duarte, California 91010, USA; 30 The Ohio State University Comprehensive Cancer Center, Columbus, Ohio 43210, USA; 31 Alliance for Clinical Trials in Oncology Statistics and Data Center, Mayo Clinic, Rochester, Minnesota 55905, USA; 32 University of Chicago Medical Center, Chicago, Illinois 60637, USA; 33 Department of Leukemia, The University of Texas MD Anderson Cancer Center, Houston, Texas 77030, USA; 34 Cancer Center, Montefiore Medical Center North Division, Bronx, New York 10467, USA; 35 Department of Pediatrics, Benioff Children's Hospital, San Francisco, California 94158, USA; 36 Helen Diller Family Comprehensive Cancer Center, San Francisco, California 94115, USA; 37 Children's Hospital of Philadelphia, Philadelphia, Pennsylvania 19104, USA; 38 Perelman School of Medicine at the University of Pennsylvania, Philadelphia, Pennsylvania 19104, USA

## Abstract

Chromosomal rearrangements are initiating events in acute lymphoblastic leukaemia (ALL). Here using RNA sequencing of 560 ALL cases, we identify rearrangements between *MEF2D* (myocyte enhancer factor 2D) and five genes (*BCL9*, *CSF1R*, *DAZAP1*, *HNRNPUL1* and *SS18*) in 22 B progenitor ALL (B-ALL) cases with a distinct gene expression profile, the most common of which is *MEF2D-BCL9*. Examination of an extended cohort of 1,164 B-ALL cases identified 30 cases with *MEF2D* rearrangements, which include an additional fusion partner, *FOXJ2*; thus, *MEF2D-*rearranged cases comprise 5.3% of cases lacking recurring alterations. *MEF2D-*rearranged ALL is characterized by a distinct immunophenotype, DNA copy number alterations at the rearrangement sites, older diagnosis age and poor outcome. The rearrangements result in enhanced MEF2D transcriptional activity, lymphoid transformation, activation of *HDAC9* expression and sensitive to histone deacetylase inhibitor treatment. Thus, *MEF2D-*rearranged ALL represents a distinct form of high-risk leukaemia, for which new therapeutic approaches should be considered.

## Abstract (web summary)

Acute lymphoblastic leukaemia is characterized by chromosomal rearrangements. Here, the authors carry out RNA-sequencing on a large cohort of patients and identify recurrent rearrangements of *MEF2D*, which lead to increased transcriptional activity of the gene, and cellular transformation *in vitro*.

## Graphical abstract

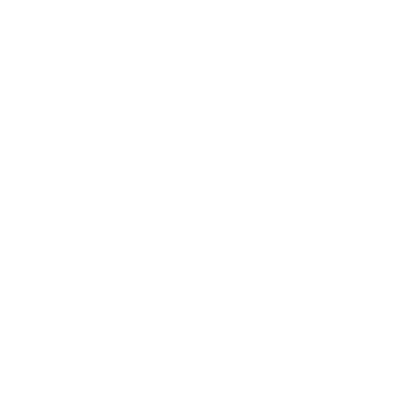

B progenitor acute lymphoblastic leukaemia (B-ALL) is characterized by recurrent gross chromosomal alterations, including aneuploidy and rearrangements that deregulate oncogenes or result in the formation of chimeric fusion proteins that have functional properties distinct from their non-rearranged counterparts. Such fusion proteins, including ETV6-RUNX1 and TCF3-PBX1, and those involving PAX5, KMT2A (MLL) and tyrosine kinases such as BCR-ABL1, are typically acquired early in leukaemogenesis and drive tumour formation by perturbing cellular pathways including haematopoietic development, tumour suppression, kinase signalling and chromatin remodelling^1^,^2^. Identification of these rearrangements is important in the management of ALL, as several are associated with treatment outcome and/or may serve as therapeutic targets.

The prevalence of recurring rearrangements varies significantly according to age and in part explains the inferior outcome of ALL in older individuals^3^,^4^. For example, rearrangements associated with favourable outcome such as *ETV6-RUNX1* are frequent in younger children compared with older children, whereas *BCR-ABL1* and rearrangements involving *CRLF2* and tyrosine kinases observed in Philadelphia chromosome-like (Ph-like) ALL are more common with increasing age^3^. However, the genetic basis of up to 30% of ALL cases is unknown.

To identify chromosomal rearrangements in B-ALL cases lacking a known recurring sentinel alteration, we performed transcriptome sequencing (RNA sequencing (RNAseq)) of 560 cases and recurrence testing in a cohort of 1,164 cases. We identified multiple new targets of rearrangement, with rearrangements of *MEF2D* and *ZNF384* defining subtypes with distinct transcriptional signatures. In total, a group of 42 cases are discovered with *MEF2D* rearranged to *BCL9*, *CSF1R*, *DAZAP1* (DAZ (deleted in azoospermia)-associated protein 1), *HNRNPUL1* (heterogeneous nuclear ribonucleoprotein U-like 1), *SS18* (synovial sarcoma translocation, chromosome 18) or *FOXJ2* (Forkhead Box J2). These rearrangements dysregulate expression of *MEF2D* target genes and, by increasing expression of *HDAC9*, create a vulnerability to targeting with histone deacetylase (HDAC) inhibitors.

## Results

### RNAseq identifies *MEF2D* fusions in a novel B-ALL subgroup

The 560 cases examined by RNAseq enrolled on the Children's Oncology Group (COG) trials included AALL0232 (*N*=216), AALL0331 (*N*=65), AALL0932 (*N*=61), AALL1131 (*N*=92), P9906 (*N*=1) and adults (age of diagnosis ≥18, *N*=122) or children (age of diagnosis <18, *N*=3) with B-ALL enroled on various non-COG protocols (Supplementary Data 1).

**Figure 1:**
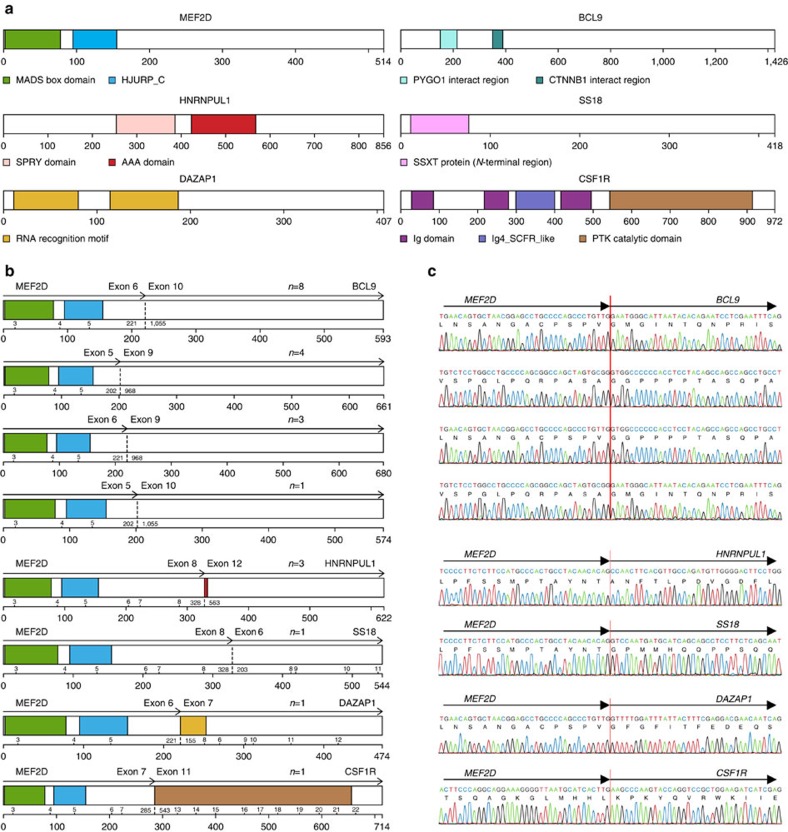
*MEF2D* rearrangements in ALL. (**a**) Protein structure of wild-type MEF2D and fusion partners, (**b**) protein structure of each fusion protein and (**c**) electropherograms showing the results of RT–PCR and Sanger sequence (see also Supplementary Fig. 1). Four *MEF2D-BCL9* isoforms were identified in 16 cases, with breakpoints involving exons 5 or 6 of *MEF2D* fused in frame to exons 9 or 10 of *BCL9*. Rearrangements involving *MEF2D* and other four fusion partners were identified in six samples. *MEF2D-DAZAP1* and *MEF2D-CSF1R* fusions have previously been reported^3^,^26^. Protein region annotation: MADS box, MEF2 (myocyte enhancer factor 2)-like/Type II subfamily of MADS; HJURP_C, Holliday junction regulator protein family *C*-terminal repeat. Ig domain, immunoglobulin domain; Ig4_SCFR_like, fourth Ig-like domain of stem cell factor receptor; PTK catalytic domain, tyrosine kinase catalytic domain.

This analysis identified multiple new recurring targets of rearrangement, including *MEF2D*, *NUTM1* and *ZNF384*, and new partners of rearrangement with known genes such as *PAX5* and *IGH* (Supplementary Data 1). We identified 22 cases (3.9%) with rearrangements of *MEF2D* at chromosome 1q21–22, encoding myocyte enhancer factor 2D (Table 1, Fig. 1, Supplementary Data 2 and Supplementary Table 1), representing 15 of 367 (4.1%) childhood cases (up to age 15 years), 4 of 62 (6.5%) adolescent cases (age 16–20 years), 2 of 73 (2.7%) young adults and a single case in 56 older adults. Five *MEF2D* fusion partners were identified: *BCL9* (16 cases), *HNRNPUL1* (3 cases), *DAZAP1*, *CSF1R* and *SS18* (1 case each). Each fusion was confirmed by reverse transcription and PCR (RT–PCR) followed by Sanger sequencing (Fig. 1, Supplementary Fig. 1 and Supplementary Table 2). In each case, the amino terminus of MEF2D was fused in frame with the carboxy-terminal portion encoded by the partner gene.

The most common *MEF2D* rearrangement was *MEF2D-BCL9*, which was present in 16 cases. Four isoforms of this fusion were identified, involving exons 5 or 6 of *MEF2D* (NM_001271629) fused in-frame to exons 9 or 10 of *BCL9* (NM_004326; Supplementary Data 2). *MEF2D-BCL9* rearrangement was associated with deregulated expression of the *C* terminus of *BCL9* distal to the rearrangement breakpoint (Supplementary Fig. 2). No fusions deregulating expression of full-length *BCL9*, such as *IGH-BCL9* (ref. 5), were identified. Only one isoform of the *MEF2D-HNRNPUL1* fusion was identified, involving exon 8 of *MEF2D* and exon 12 of *HNRNPUL1*. Expression of the fusion proteins in patient leukaemic cells was confirmed by immunoblotting (Supplementary Fig. 3) and the disruption of MEF2D and MEF2D-BCL9 rearrangements were shown to be present in the majority of leukaemic cells by fluorescence *in situ* hybridization (FISH), consistent with the fusions being leukaemia-initiating events acquired early in leukaemogenesis (Supplementary Fig. 4). Several patterns of probe hybridization were identified, consistent with diverse mechanisms of rearrangement, including insertion of the fusion into a different chromosome (for example, SJBALL020667), interstitial insertion at chromosome 1q (SJBALL020703) and reciprocal rearrangement (SJBALL020987).

### Genomic alterations of *MEF2D*-rearranged cases

*MEF2D* and *BCL9* are both located at 1q21.2–22 and the common pattern of interstitial insertion resulted in these rearrangements being cryptic on cytogenetic analysis. We observed that many rearrangements were accompanied by copy number alterations at the *MEF2D* and partner gene loci. Ten cases harbouring *MEF2D* rearrangements had single-nucleotide polymorphism (SNP) microarray data available (Table 1), with eight of ten exhibiting focal gains or loss of DNA at the partner genes (*MEF2D*, *BCL9*, *DAZAP1* and *HNRNPUL1*; Supplementary Fig. 5). The genomic structure of the *MEF2D-BCL9* rearrangements was confirmed in two cases by whole genome sequencing (Supplementary Fig. 6).

Additional genetic alterations identified in *MEF2D*-rearranged cases included *IKZF1* alterations in three cases, including exons 4–8 deletions in two cases and exons 4–7 deletion in one case (resulting in the expression of the dominant negative IK6 isoform). Mutational analysis of RNAseq data showed that 6 of 16 *MEF2D-BCL9* cases harboured activating *NRAS* mutations, although these were frequently subclonal and thus secondary events in leukaemogenesis (Supplementary Data 1 and Supplementary Table 3). Analysis of Ras mutations (*NRAS*, *KRAS*, *NF1* and *PTPN11*) across the RNAseq cohort revealed a similar prevalence of Ras mutations as other subtypes of ALL previously reported to be enriched for Ras mutations (for example, hyperdiploid and *MLL*-rearranged ALL (Supplementary Fig. 7)).

### The transcriptional signatures of *MEF2D* and *ZNF384* ALL

**Figure 2:**
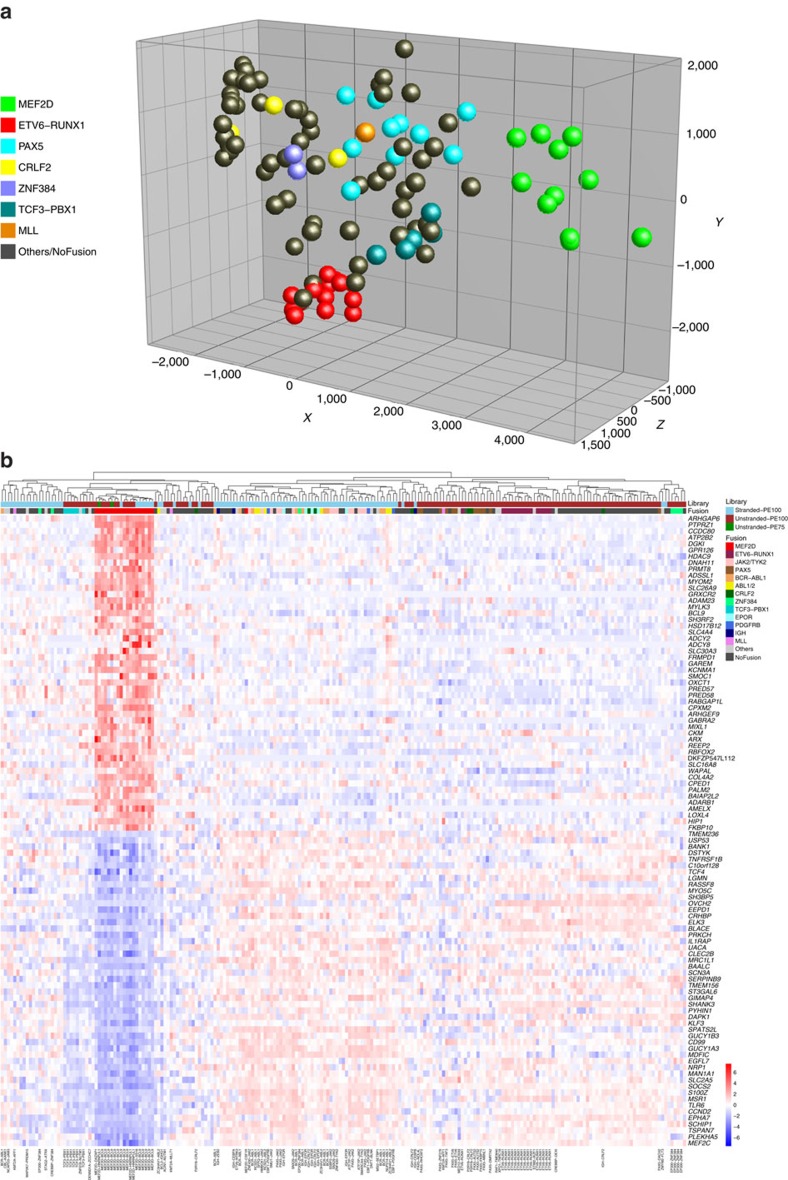
*MEF2D-*rearranged ALL cases have a distinct gene expression profile. (**a**) Principal component analysis of samples subjected to unstranded RNAseq data, showing clustering of *MEF2D-*rearranged cases. A full list of cases and rearrangements identified by RNAseq is provided in Supplementary Data 1. (**b**) The gene expression signature for *MEF2D*-rearranged ALL was determined by comparing 19 *MEF2D* fusion samples (excluding 2 cases that failed quality-control threshold for gene expression analysis and one *MEF2D-CSF1R* rearranged case) and 199 control samples through Wald's test and Benjamini–Hochberg adjustment. Genes with adjusted *P*-value <0.01 are provided in Supplementary Data 3. The top 50 upregulated and top 50 downregulated genes (based on the adjusted *P*-value) were selected to perform supervised clustering and shown in the order from most significant upregulated genes (top) to the most downregulated ones (bottom). Stranded-PE100, total stranded paired-end 100 bp RNAseq; unstranded-PE100, unstranded mRNA paired-end 100 bp RNAseq; unstranded-PE75, unstranded mRNA paired-end 75 bp RNAseq. MEF2D, fusions involving *MEF2D*; JAK2/TYK2, fusions involving *JAK2* or *TYK2*; PAX5, fusions with *PAX5* as the *N*-terminal fusion partner with non-kinase genes; ABL1/2, fusions with *ABL1* or *ABL2* observed in Ph-like ALL; CRLF2, *IGH-CRLF2* or *P2RY8-CRLF2* fusions; ZNF384, fusions involving *ZNF384* as the *C*-terminal fusion partner; EPOR, fusions deregulating *EPOR* observed in Ph-like ALL; PDGFRB, fusions involving *PDGFRB*; IGH, *IGH* fusions except *IGH-CRLF2* and *IGH-EPOR*; MLL, fusions involving *KMT2A*(*MLL*) gene.

Although RNAseq data were obtained from diverse library preparation (total versus messenger RNA, unstranded versus stranded) and sequencing (75 bp versus 100 bp paired end) methods, reproducible clustering of *MEF2D-*rearranged cases was observed in principal component analysis and hierarchical clustering (Fig. 2a and Supplementary Figs 8–10), with the exception of one sample harbouring an *MEF2D-CSF1R* fusion, which clustered with Ph-like ALL cases. Rearrangements of *CSF1R*, encoding the macrophage colony-stimulating factor receptor, are observed in Ph-like ALL and result in activation of tyrosine kinase signalling pathways inhibited by kinase inhibitors such as imatinib and dasatinib^3^,^6^,^7^. Thus, the clustering of this sample with other Ph-like ALL cases distinct from other *MEF2D-*rearranged cases was not surprising. Supervised analysis of *MEF2D-*rearranged cases identified a distinct gene expression signature with substantial overlap between cases sequenced using stranded and unstranded sequencing methods (Fig. 2b and Supplementary Data 3–5).

Analysis of outlier gene expression in high-risk childhood ALL previously identified eight ALL subgroups, several of which harboured recurring genetic alterations including rearrangement of *KMT2A* (*MLL*) (ROSE group 1, R1), *TCF3-PBX1* (R2), deregulation of *ERG* (R6) and Ph+/Ph-like ALL (R8), and so on^8^. Analysis of 1,164 childhood, adolescent and young adult ALL cases with available microarray gene expression data identified 31 cases in the R3 subgroup that lacked known chromosomal rearrangements (Supplementary Fig. 11), 10 of which were in the discovery RNAseq cohort and had *MEF2D* rearrangements (Supplementary Data 1). RT–PCR identified known *MEF2D* rearrangements in 19 of the 20 remaining cases (Supplementary Data 6 and Supplementary Fig. 12). One of the two remaining cases had sufficient material for RNAseq, which identified a novel chimeric in-frame fusion of *MEF2D* exon 8 to exon 7 of the forkhead transcription factor gene, *FOXJ2* (Supplementary Data 6). Taken together, 42 *MEF2D*-rearranged cases were discovered in this study that exhibit a distinct gene expression profile. Moreover, RNAseq identified 20 cases with rearrangements of *ZNF384* to 6 different partners (*ARID1B*, *CREBBP*, *EP300*, *SMARCA2*, *TAF15* and *TCF3*), which were also associated with a distinct gene expression profilez and exclusively observed in the R5 group (Supplementary Data 7 and Supplementary Fig. 13). Together, these findings suggest that *MEF2D* and *ZNF384* rearrangements are founding alterations of the R3 and R5 subgroups, respectively, and represent distinct subtypes of B-ALL.

To explore the deregulated gene expression characteristics of *MEF2D*-rearranged ALL, we perform the gene set enrichment analysis (GSEA) and pathway analysis and demonstrated that *MEF2D-*rearranged cases exhibited a gene expression profile of a later maturational stage than other subtypes of ALL (Supplementary Data 8 and 9). We did observed negative enrichment of WNT signalling, consistent with the observation that the *MEF2D-BCL9* fusion removes the β-catenin-interacting domains of BCL9. The clustering of gene expression profiles *MEF2D-*rearranged cases irrespective of *C*-terminal fusion partner suggests that deregulation of MEF2D function may in part mediate leukaemogenesis. To examine this, we compared the gene expression profile of *MEF2D-*rearranged ALL with existing chromatin immunoprecipitation and sequencing and RNAseq data sets for *MEF2D* studies in muscle, retinal and lymphoid cells, and observed partial overlap with known MEF2D-bound genes. Notably, *HDAC9*, one of the top upregulated genes was consistently represented in the data sets (Supplementary Data 10). *HDAC9* is a direct transcriptional target of MEF2 and acts to inhibit the transcriptional activity of MEF2C^9^, consistent with the striking downregulation of this gene in *MEF2D-*rearranged ALL (Supplementary Data 3–5 and Supplementary Figs 14 and 15).

### Clinical and pathological features of *MEF2D* ALL

The median age of *MEF2D*-rearranged cases at diagnosis was 14.0 years (range 4.8–48.0) for the 42 *MEF2D* rearranged cases and 14.0 years (range 4.8–21.3) for 29 *MEF2D-BCL9* cases with approximately equal occurrence in male and female patients (Supplementary Data 1 and 6). The immunophenotype of *MEF2D-*rearranged ALL was distinct from other subtypes of B-ALL and was characterized by weak or absent expression of CD10 and high expression of CD38. Low or absent expression of CD10 is a feature of *MLL-*rearranged ALL and high CD38 expression is typically seen in normal, regenerating lymphoid cells (haematogones), but these features are otherwise uncommon in B-cell precursor ALL (Supplementary Table 4).

**Figure 3:**
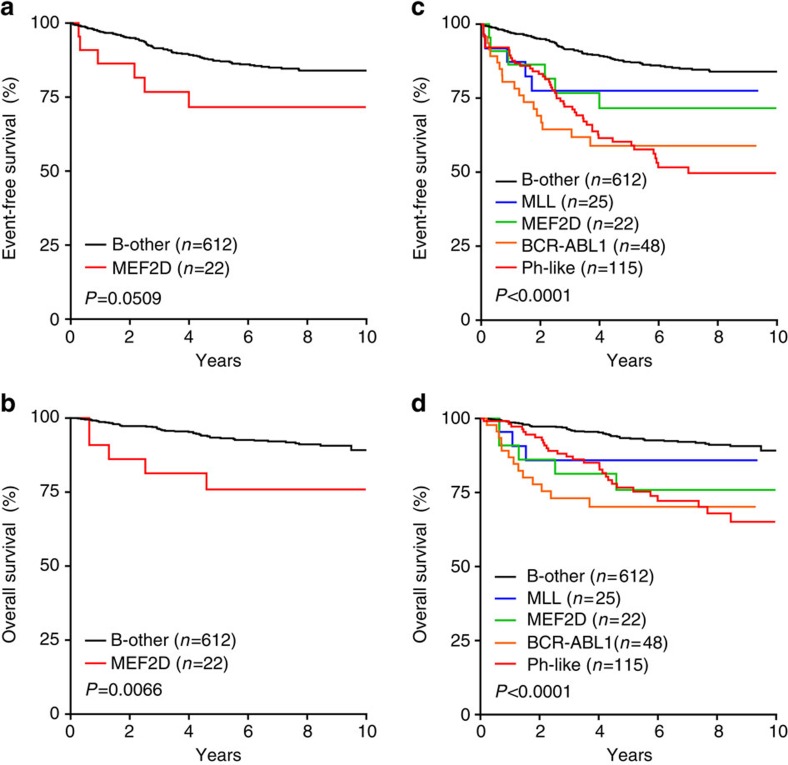
Poor outcome of *MEF2D-*rearranged ALL. The event-free (**a**,**c**) and overall survival (**b**,**d**) outcome of *MEF2D-*rearranged cases is compared with that of B-precursor ALL cases lacking known recurring chromosomal rearrangements (**a**,**b**) and key genetic subtypes (**c**,**d**) of B-ALL in the COG AALL0232 study.

The outcome of *MEF2D-*rearranged ALL was inferior to that of other ALL subtypes. Although the number of *MEF2D-*rearranged cases was small compared with several other subtypes, analysis of children with ALL enroled on the AALL0232 study of high-risk pre-B ALL showed that the 5-year event-free survival (EFS) of *MEF2D-*rearranged ALL was 71.6% (s.e.±10.2%, *n*=22), compared with that of *BCR-ABL1* (59.9±8.9%, *n*=48), *MLL* (77.5±10.6%, *n*=25), Ph-like (60.3±5.5%, *n*=115) and other pre-B ALL cases (87.3±1.6%, *n*=612; log rank test *P*<0.0001; Fig. 3). Notably, Ras mutations were not associated with inferior outcome in this cohort (5 year EFS 89.7±4.2% for 62 cases with Ras mutations compared with 76.2±4.4% for 112 cases without, log rank test, *P*=NS). Through multivariable analysis with the Cox proportional-hazards regression model^10^, *MEF2D* rearrangements were not significantly associated with poor outcome following correction for age (≥10 years unfavourable, *P*=0.0034), MRD positivity (*P*<0.0001), peripheral blood leukocyte count (≥100 mm^−3^ unfavourable, *P*=0.0067) and sex (*P*=NS). Further analysis of larger cohorts of *MEF2D-*rearranged cases will be required to determine whether this subset is independently associated with poor outcome.

### Deregulation of expression of MEF2D target genes

**Figure 4:**
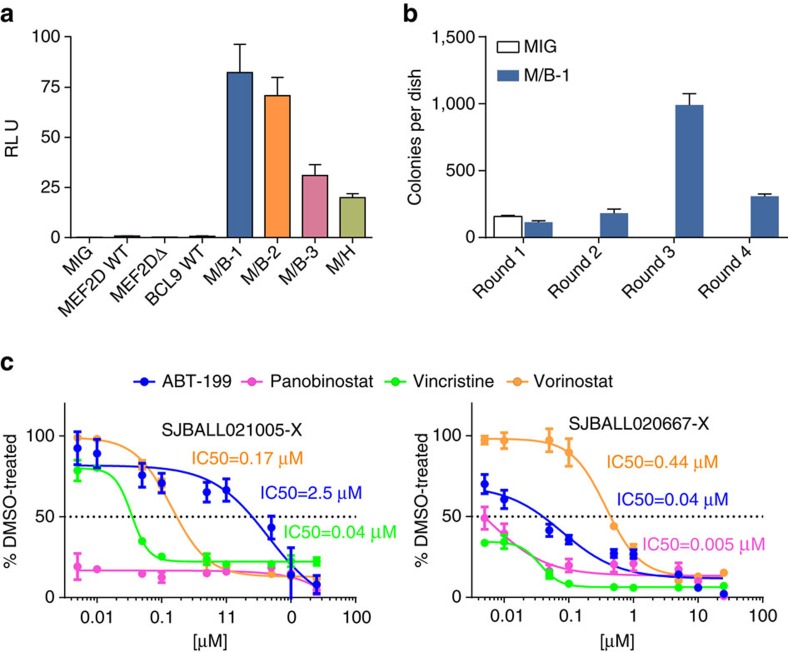
Luciferase and drug-sensitivity assays. (**a**) Luciferase assay to test MEF2 binding activities. The average levels of normalized luciferase activities±s.d. from six replicates are indicated. MEF2D-rearranged isoforms are more potent transcriptional activators than wild-type and truncated MEF2D. (**b**). Colony-forming assay for retroviral vectors transduced lineage negative bone marrow cells. Colony number counted from three replicate dishes is shown in mean±s.d. Western blotting was performed to verify expression of proteins by each construct (Supplementary Fig. 18). RLU, relative luminescence units; MIG, MSCV-IRES-GFP, represents empty vector; MEF2D-WT, vectors express wild-type MEF2D; MEF2DΔ, truncated MEF2D; BCL9-WT, wild-type BCL9; M/B-1 through M/B-3, three most common *MEF2D-BCL9* fusion isoforms; M/H, *MEF2D-HNRNPUL1*. (**c**) *Ex vivo* drug sensitivity assays from two xenografted samples. Four chemical compounds including ABT-199 (selective BCL-2 inhibitor), vincristine and two HDAC inhibitors (panobinostat and vorinostat) were applied to measure growth inhibition curves in xenografted samples. The *MEF2D* rearrangements were confirmed in xenografted samples by RT–PCR and three more xenografted samples were also tested by the four drugs (Supplementary Fig. 16). Error bars represent mean±s.d.

The clustering of gene expression profiling data of *MEF2D-*rearranged cases with this diverse range of partner genes strongly suggests the fusions exert similar oncogenic effects. All fusions preserve the MEF2D MADS-box domain that mediates DNA binding and potentially dimerization. Thus, aberrant function mediated by MEF2D transcriptional activation is likely to be central in leukaemogenesis. To examine this, we performed transcriptional activation assays in which the ability of MEF2D fusion proteins, wild-type MEF2D and BCL9, and the rearranged *N* terminus of MEF2D to activate transcriptional targets were examined. This showed that each MEF2D fusion protein was significantly more potent in activating expression than wild-type MEF2D, and that truncated MEF2D was inactive (Fig. 4a). Thus, each fusion partner stabilizes and augments MEF2D transcriptional activation. In colony-forming assays, MEF2D-BCL9 sustained serial replating of lymphoid colonies, indicating that this fusion confers haematopoietic self-renewal (Fig. 4b).

### Sensitivity to HDAC inhibition

The poor outcome of *MEF2D*-rearranged ALL suggests that new therapeutic approaches directed against deregulated cellular pathways observed in this form of leukaemia are warranted. To explore this, we established xenografts of leukaemic cells of five cases with *MEF2D*-rearranged ALL engrafted in immunocompromised mice. Following engraftment, leukaemic cells were harvested from the bone marrow and spleen, purified and exposed to chemotherapeutic agents *ex vivo*. This demonstrated sensitivity to the HDAC inhibitor panobinostat (but little sensitivity to BCL9 inhibitors), suggesting HDAC inhibition as a therapeutic option in this high-risk form of leukaemia (Fig. 4c and Supplementary Fig. 16a–d). Although HDAC inhibitors such as panobinostat are broadly active in many haematopoietic tumours, including ALL^11^ (data for non-MEF2D ALL subtypes are shown in Supplementary Fig. 17) and are being explored as a therapeutic option for a range of relapsed and refractory tumours, we observed exquisite sensitivity in MEF2D-rearranged ALL (Fig. 4c). In contrast, and consistent with a lack of activation of β-catenin signalling from *MEF2D-BCL9* fusions, we observed a lack of efficacy of selective, potent BCL9 inhibitors^12^ *ex vivo* (Supplementary Fig. 16e–h).

## Discussion

The distinct gene expression profile, tumour cell immunophenotype, older age of onset and poor outcome of *MEF2D-*rearranged ALL cases together suggest that *MEF2D-*rearranged ALL represents a biologically distinct form of leukaemia. The fusion partners of *MEF2D* have a diverse range of biological functions. MEF2D is a member of a family of four myocyte enhancer factor transcription factors with an important role in neuronal differentiation^13^,^14^. MEF2D is expressed throughout B-cell differentiation^15^ and inactivation of *Mef2c/d* results in an arrest in B-lymphoid maturation at the pre-B-cell stage^15^,^16^. *BCL9* is located on chromosome 1q21 and was first identified as the target of a t(1;14)(q21;q32) *IGH-BCL9* rearrangement in the pre-B-cell leukaemia cell line CEMO-1 (refs 5, 17). Although *IGH* is rearranged to a variety of cytokine receptors and transcription factor genes in ALL, including *CRLF2*, *EPOR* and members of the CEBP family of transcription factors^18^,^19^,^20^,^21^, and multiple known and novel *IGH* rearrangements, were identified (Supplementary Data 1), none of the *BCL9* rearrangements identified in this study involved *IGH.* BCL9 is a component of the WNT/β-catenin signalling cascade that has important roles in development, stem cell self-renewal and oncogenesis. BCL9 is part of the nuclear complex consisting of T-cell factor/lymphoid enhancer factor, β-catenin, BCL9/BCL9L and PYGO, which activates transcription of canonical WNT target genes such as *FGF20*, *DKK1*, *WISP1*, *MYC*, *CCND1* and *GCG* (Glucagon)^22^,^23^. BCL9 has also been shown to have an oncogenic role in multiple myeloma^24^.

The *MEF2D-BCL9* rearrangement results in deregulated expression of the *C* terminus *BCL9* expression (Supplementary Fig. 2), raising the possibility that these fusions may perturb WNT/β-catenin signalling. However, the *MEF2D-BCL9* rearrangements involve only the last one or two of ten exons of *BCL9* exons resulting in loss of the BCL9 domains that mediate interaction with PYGO1 and β-catenin, consistent with the lack of enrichment of WNT/β-catenin signalling in the gene expression profile of *MEF2D-*rearranged ALL. Thus, *IGH-BCL9* and *MEF2D-BCL9* probably exert different roles in leukaemic transformation. *DAZAP1* is located at 19p13.3 and encodes an RNA-binding protein that interacts with the infertility factors DAZ and DAZL, and other RNA-binding proteins^25^. The *MEF2D-DAZAP1* rearrangement has previously been identified and shown to result in proliferation of fibroblasts^26^,^27^. *HNRNPUL1* encodes a nuclear RNA-binding protein of the heterogeneous nuclear ribonucleoprotein family that may exert a role in nucleocytoplasmic RNA transport and DNA repair^28^. *SS18* is fused with *SSX1* or *SSX2* in synovial sarcoma, and has putative roles in chromatin remodelling and WNT/β-catenin signalling^29^,^30^. *FOXJ2* is a transcription factor that has been reported to affect migration and invasiveness of various tumours^31^,^32^ but, unlike other forkhead transcription factor genes, has not previously been identified as a target of rearrangement in cancer.

These data demonstrate the power of transcriptome sequencing to identify novel rearrangements that define new genetic subtypes of ALL. The finding of *MEF2D* rearrangements as a hallmark of a subtype of ALL with a distinct gene expression profile provides another example of ALL subtypes that are cryptic (in this case, due to the common involvement of rearrangement of two genes co-located on one chromosome) and represented by rearrangement to diverse partners rather than a single fusion observed in other subtypes of ALL. Despite the common involvement of the WNT co-factor BCL9, there was no evidence of dysregulated WNT/β-catenin signalling in leukaemogenesis. In contrast, deregulated MEF2D activity, with activation of *HDAC9* and resulting inhibition of MEF2C were characteristic of this form of leukaemia. Further studies are required to dissect the relative role of MEF2D and partner gene involvement in leukaemogenesis, but our existing data suggest the potential for HDAC inhibition in this form of leukaemia.

## Methods

### Patients and samples

Patients with ALL were enroled on Alliance—Cancer and Leukemia Group B, COG, City of Hope, Eastern Cooperative Oncology Group, MD Anderson Cancer Center, Medical Research Council UK, Northern Italian Leukemia Group, Southwestern Oncology Group and University of Toronto Protocols (Supplementary Data 1). Patients and/or guardians provided informed consent/assent. The study was approved by the St. Jude Children's Research Hospital Institutional Review Board. Leukaemic cells from bone marrow aspirates obtained at diagnosis were processed by density gradient centrifugation. Samples of <70% tumour cell content were purified by fluorescence-activated cell sorting before RNA extraction.

### Transcriptome sequencing and data analysis

Transcriptome sequencing was performed using TruSeq library preparation and HiSeq 2000 and 2500 sequencers. All sequencing was paired end and was performed using (1) total RNA and stranded RNA sequencing (100 bp reads), and (2) polyA-selected mRNA (100 or 75 bp reads). Sequencing reads were mapped to the GRCh37 human genome reference by STAR^33^ (version 2.5.1b) through the two pass mapping pipeline. Gene annotation downloaded from Ensembl website (http://www.ensembl.org/) was used for STAR mapping and the following reads count evaluation. Cicero^3^ and FusionCatcher^34^,^35^ were used to detect fusions.

To assess gene expression profile, reads count for each annotated gene was extracted by HTSeq package^36^ and gene expression level normalization and differential expression analysis was carried out by using DESeq2 bioconductor R package^37^. To evaluate the relative gene expression level, regularized log-transformed (rlog) value was calculated by DESeq2.

Gene expression profile between samples with and without *MEF2D* fusions were examined in two ALL cohorts. These included a cohort of total stranded RNAseq (30-fold coverage on exon regions (D30C)>20%) and cases with unstranded mRNA sequencing (D30C>30%). Two *MEF2D*-rearranged cases failed to meet the requirement of D30C>20% were excluded, both of which were sequenced by unstranded 75 bp paired end protocol. Two hundred and nineteen samples were available for gene expression profiling analysis, including 95 samples with total stranded RNAseq (this cohort was enriched for Ph-like ALL and included 8 cases with *MEF2D* fusions) and 124 samples with unstranded RNAseq (enriched for other B-ALL and included 12 cases with *MEF2D* fusions) data are available for the gene expression profiling and gene signature analysis.

### GSEA and pathway analysis

Normalized gene expression levels were evaluated from RNAseq data and subjected to DESeq2 for differential gene expression analysis. As recommended from GSEA^38^, genes ranked according to the significance of differential gene expression levels between samples with and without target rearrangements and then input to GSEA to calculate potentially enriched gene sets from a homemade gene sets database. To assess the pathway enrichment, genes with *P*-value ≤0.01 and fold change ≥2 were selected to run DAVID pathway analysis^39^.

### Mutation detection from RNAseq data

The mutations were called according the GATK^40^ forum recommended pipeline for calling variant in RNAseq data (http://gatkforums.broadinstitute.org/gatk/discussion/3891/calling-variants-in-rnaseq). Specifically, STAR mapped bam files were processed by Picard (http://broadinstitute.github.io/picard) to mark duplicate reads, then a GATK module SplitNCigarReads was used to splits reads into exon segments and hard-clip any sequences overhanging into the intronic regions. Variant calling was performed by the HaplotypeCaller module in GATK and then the variants were quality controlled by the following steps: (1) at least three reads support the mutation and the mutant allele frequency is ≥0.05; (2) not observed in common SNP database (from UCSC version 142); and (3) not observed in ≥2 samples from our germline exome sequence cohort (775 samples). After filtering, all the mutations were annotated to genes according to their genomic positions. Non-silent mutations were compared with COSMIC somatic mutation database (GRCh37-V74) and the overlapped cancer relevant genes (http://cancer.sanger.ac.uk/census) were kept for further analysis.

### Retroviral constructs and immunoblotting

*MEF2D*-rearranged isoforms, wild-type *MEF2D* and *BCL9* were amplified by RT–PCR using Phusion High-Fidelity DNA Polymerase (M0530L, New England Biolabs, Inc.) and primers (Supplementary Table 5) from either leukaemic cell or MOLT4 cell line complementary DNA. Amplification products were purified by Wizard SV Gel and PCR Clean-up system (Promega) and verified by Sanger sequencing. Purified PCR products were cloned into pCR-Blunt II-TOPO (Life Technologies) and sub-cloned into the murine stem cell virus-internal ribosome entry site-green fluorescent protein (MSCV-IRES-GFP) retroviral vector.

Truncated *MEF2D* isoform was amplified from MSCV*-MEF2D-BCL9*-IRES-GFP vector (M/B-1 vector, the one with the most common *MEF2D-BCL9* fusion isoform) by the same PCR system and specially designed primers for Gateway cloning (Vector NTI, Thermo Fisher) (Supplementary Table 5). Primers for truncated *MEF2D* were designed to amplify from the *N* terminus of *MEF2D* to the last amino acid fused to BCL9 with a stop codon inserted at the fusion point. Purified PCR products were cloned into the Gateway pDONR221 vector (Thermo Fisher) by BP Clonase Enzyme (Thermo Fisher). Truncated *MEF2D*-pDONR221 vector was shuttled into a Gateway-compatible MSCV-IRES-GFP vector using the LR Clonase enzyme (Thermo Fisher). Constructs were verified by Sanger sequencing (Supplementary Table 5).

Transfected/transduced cells and human leukaemic cells were lysed in RIPA buffer supplemented with phosphatase inhibitors (Sigma) and 30 μg protein of patient samples (15 μg of lysate from NIH-3T3 cells) electrophoresed through 4–12% NuPage Bis-Tris gels (Life Technologies) at 190 V for 80 min. Blots were probed with anti-MEF2D (H-57, sc-366368), anti-actin (I-19) (both, Santa Cruz Technologies) and anti-BCL9 (Abcam ab54833). For human leukaemic cells, 1:250, 1:500 and 1:2,000 dilutions were used for MEF2D, BCL9 and β-actin antibodies, respectively; for transfected/transduced cells, the 3 antibodies were 1:1,000 diluted. Five percent BSA in Tris-buffered saline with 0.1% Tween 20 solution was used to dilute the antibodies.

### Luciferase assays

In 96-well dishes, 2 × 10^4^ 293T cells were transfected with 0.04 pmols of MSCV-IRES-GFP (MIG) vectors expression wild-type MEF2D, truncated MEF2D (MEF2DΔ), wild-type BCL9, three most common MEF2D-BCL9 fusion isoforms, MEF2D-HNRPULL1 or empty vector along with 250 ng of a 3XMEF2 firefly luciferase reporter construct (Addgene plasmid #32967) and 50 ng of a pRL-TK *Renilla* luciferase reporter plasmid (Promega) using FuGene HD (Roche Diagnostics). Forty-eight hours after transfection, measurement of firefly and *Renilla* luciferase activity was performed using the Dual-Glo Luciferase Assay System (Promega E2920) according to the manufacturer's instructions. All transfections were performed with six technical replicates in two independent experiments. Firefly luciferase activity was normalized according to corresponding *Renilla* luciferase activity and reported as TBS-T ±s.d. from one representative experiment.

### Generation of xenografted mice

One million patient-derived leukaemic cells were transplanted into three mice each by tail vein injection into 8–10-week-old sublethally irradiated (250 rads) female immunodeficient NOD.Cg-*Prkdc*^scid^*Il2rg*^*tm1Wjl*^/SzJ (NSG) mice. Disease progression was monitored by flow cytometric analysis of peripheral blood cells stained for mCD45, hCD45, hCD19 and hCD7, and the bone marrow and spleen were harvested when >80% of hCD45+ hCD19+ cells observed in peripheral blood. Mice were housed in an American Association of Laboratory Animal Care-accredited facility and were treated on Institutional Animal Care and Use Committee-approved protocols in accordance with NIH guidelines. The study was approved by institutional review board of St Jude Children's Research Hospital.

### *Ex vivo* drug sensitivity assays

Human xenograft cells were flushed from tibiae and femora of moribund mice, subjected to red cell lysis, washed and immediately plated 1 × 10^5^ per well in 96-well plates containing media with the indicated concentrations of compounds in triplicate. Cells were treated for 48 h, incubated with resazurin for 4 h and read on a Synergy HT (Biotek).

### Colony-forming assays

Bone marrow from 10-week-old C57Bl/6 wild-type mice was extracted from the tibiae and femora. Red blood cells were lysed and the remaining bone marrow cells were incubated with a cocktail of biotinylated anti-mouse antibodies (Gr-1, B220, Ly-1, Ter119, Mac-1, diluted 1:100 in PBS with 5% rat serum and 5% FCS; BD Biosciences) followed by mixing with streptavidin-coated beads (Dynabeads M-280 Streptavidin; Life Technologies). Cells were separated on a magnet and unbound cells were collected and incubated at 37 °C, 8% CO_2_ for 2 days in the presence of interleukin (IL)-3, IL-6, stem cell factor (SCF), IL-7 and Flt3 cytokines (Peprotech). Cells were retrovirally transduced with empty MSCV-IRES-GFP (MIG) and MSCV-*MEF2D-BCL9*-IRES-GFP (M/B-1) vectors on RetroNectin (Takara Bio) for 48 h before sorting for GFP-positive cells (BD FACSAria II, BD Biosciences). For clonogenic assays, 10,000 cells were plated in triplicate in Methocult M3231 (Stem Cell Technologies, Inc.) with the appropriate factors (stem cell factor, 100 ng ml^−1^; FLT-3 ligand, 10 ng ml^−1^; IL-7, 20 ng ml^−1^) and colonies were scored 7 days later. For re-plating, 10,000 cells were cultured in identical conditions, with colonies counted on day 7–10.

### FISH analysis

FISH analysis was performed to confirm chromosomal rearrangements. As previously described^3^,^41^, all assays were performed as sequential hybridization events first using the 5′- and 3′-target gene-specific probes in different colours, to demonstrate the presence or absence of intragenic disruption. Imaging was performed following the first hybridization and slide coordinates were recorded. A second hybridization was then performed using the appropriate 5′-promoter probe and a second set of images were then made of the previously imaged cells. To confirm *MEF2D-BCL9* rearrangements, sequential probing for *MEF2D* and *BCL9* was performed using probes prepared from clones RP11-16P22 (*MEF2D* 5′), RP11-959J12 (*MEF2D* 3′) and CH17-105C9 (*BCL9* 3′); to test *IGH-BCL9* fusions, sequential probing for *IGH* enhancer and *BCL9* was carried out by using probes from RP5-998D24 (*IGH* enhancer) and CH17-105C9 (*BCL9* 3′).

### Statistical analysis

Associations between ALL subtype and treatment outcome (EFS and overall survival) were performed using the Kaplan–Meier estimator with Peto's estimator of s.d. and the log-rank test^42^,^43^,^44^. Analyses were performed using Prism v6.0 (GraphPad), R (www.r-project.org)^45^ and SAS (v9.1.2, SAS Institute, Cary, NC).

### Data availability

Genome and RNAseq data are deposited at the European Genome Phenome archive, accession EGAS00001000654 (ref. 3), EGAS00001001952 and the database of Genotypes and Phenotypes (dbGaP) under accession code phs000463. Data derived from COG samples, including gene expression and SNP microarray data, may also be accessed through the Therapeutically Applicable Research to Generate Effective Treatments data matrix (https://ocg.cancer.gov/programs/target). The remaining data are available within the article and its Supplementary Files or available from the authors upon request.

## Additional information

**How to cite this article:** Gu, Z. *et al.* Genomic analyses identify recurrent *MEF2D* fusions in acute lymphoblastic leukaemia. *Nat. Commun.* **7,** 13331 doi: 10.1038/ncomms13331 (2016).

**Publisher's note:** Springer Nature remains neutral with regard to jurisdictional claims in published maps and institutional affiliations.

## Supplementary Material

Supplementary Information Supplementary Figures 1-18, Supplementary Table 1-5 and Supplementary Reference

Supplementary Data 1 Details of sample cohort.

Supplementary Data 2 Details of MEF2D fusion isoforms.

Supplementary Data 3 Gene expression signature of MEF2D-rearranged cases-stranded and unstranded.

Supplementary Data 4 Gene expression signature of MEF2D-rearranged cases-stranded only.

Supplementary Data 5 Gene expression signature of MEF2D-rearranged cases-unstranded only.

Supplementary Data 6 Confirmed MEF2D rearrangements in R3 samples.

Supplementary Data 7 Gene expression signature of ZNF384-rearranged cases.

Supplementary Data 8 Gene set enrichment analysis result.

Supplementary Data 9 Pathway analysis of the MEF2D-rearranged gene signature.

Supplementary Data 10 Overlap with public ChIPseq-RNAseq data.

## Figures and Tables

**Table 1:**
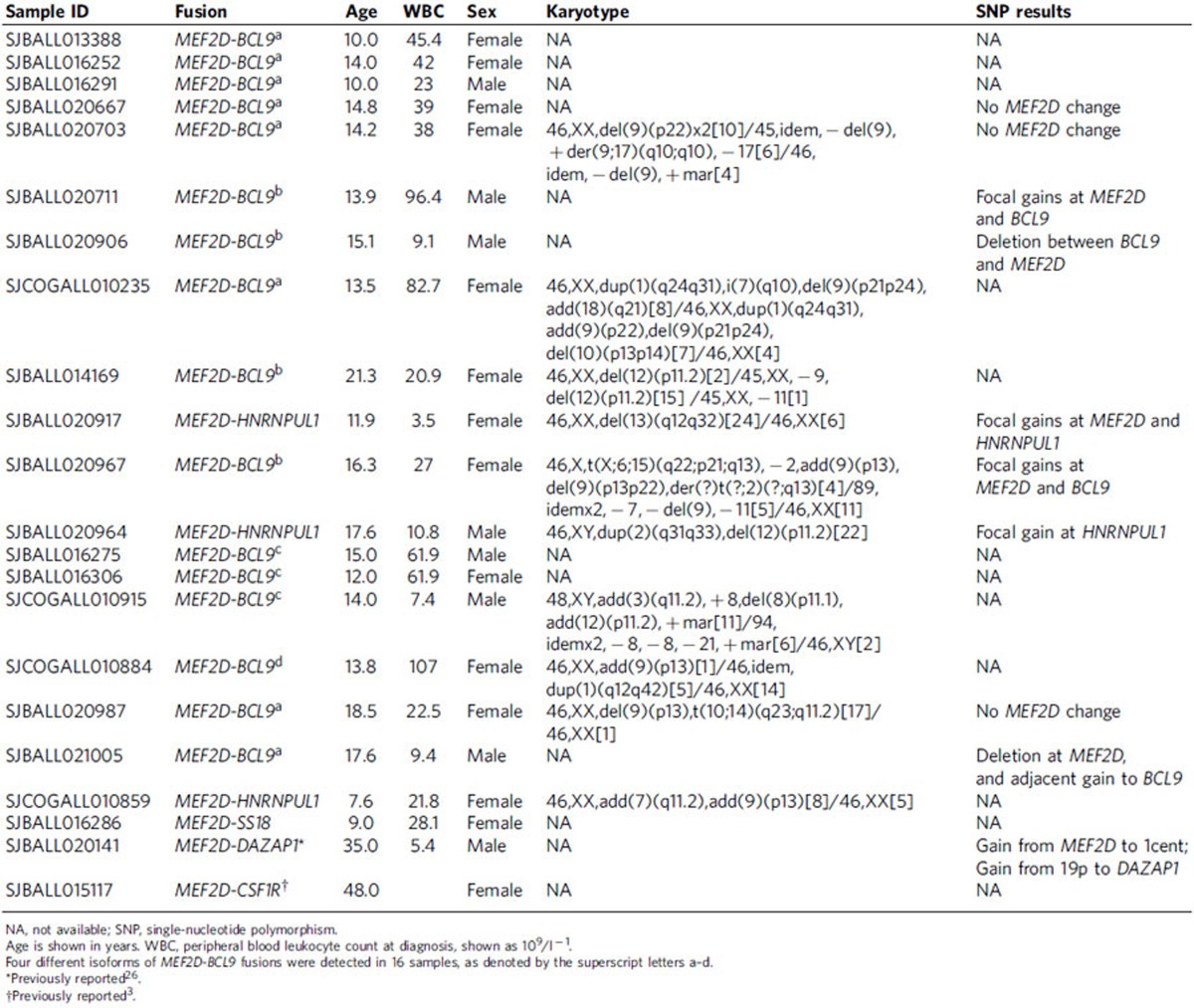
Clinical and genetic features of patients with *MEF2D*-rearranged ALL.
