# Supplementary material for: Genomic analyses identify recurrent MEF2D fusions in acute lymphoblastic leukaemia
Source: Nat Commun. 2016 Nov 8;7:13331. doi: 10.1038/ncomms13331 (PMC5105166; doi:10.1038/ncomms13331)
Supplement: Supplementary Information — Supplementary Figures 1-18, Supplementary Table 1-5 and Supplementary Reference [file ncomms13331-s1.pdf]

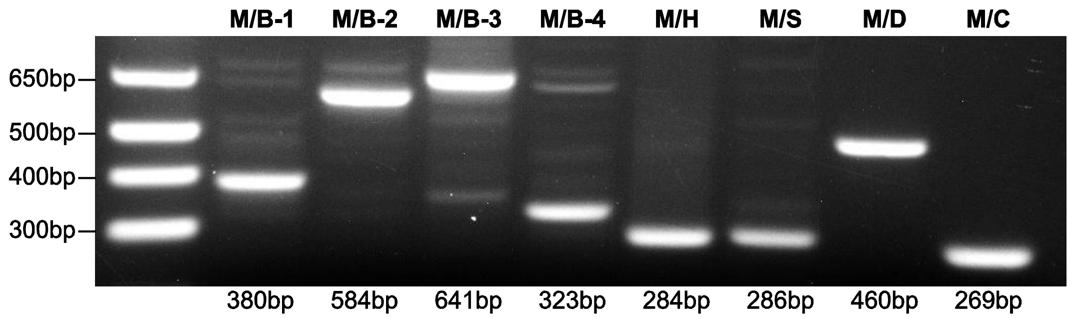

Supplementary Figure 1. *MEF2D* fusion validation by RT-PCR.

M/B-1 through M/B-4, 4 different isoforms of *MEF2D-BCL9* fusion; M/H, *MEF2D-HNRNPUL1*; M/S, *MEF2D-SS18*; M/D, *MEF2D-DAZAP1*; M/C, *MEF2D-CSF1R*.

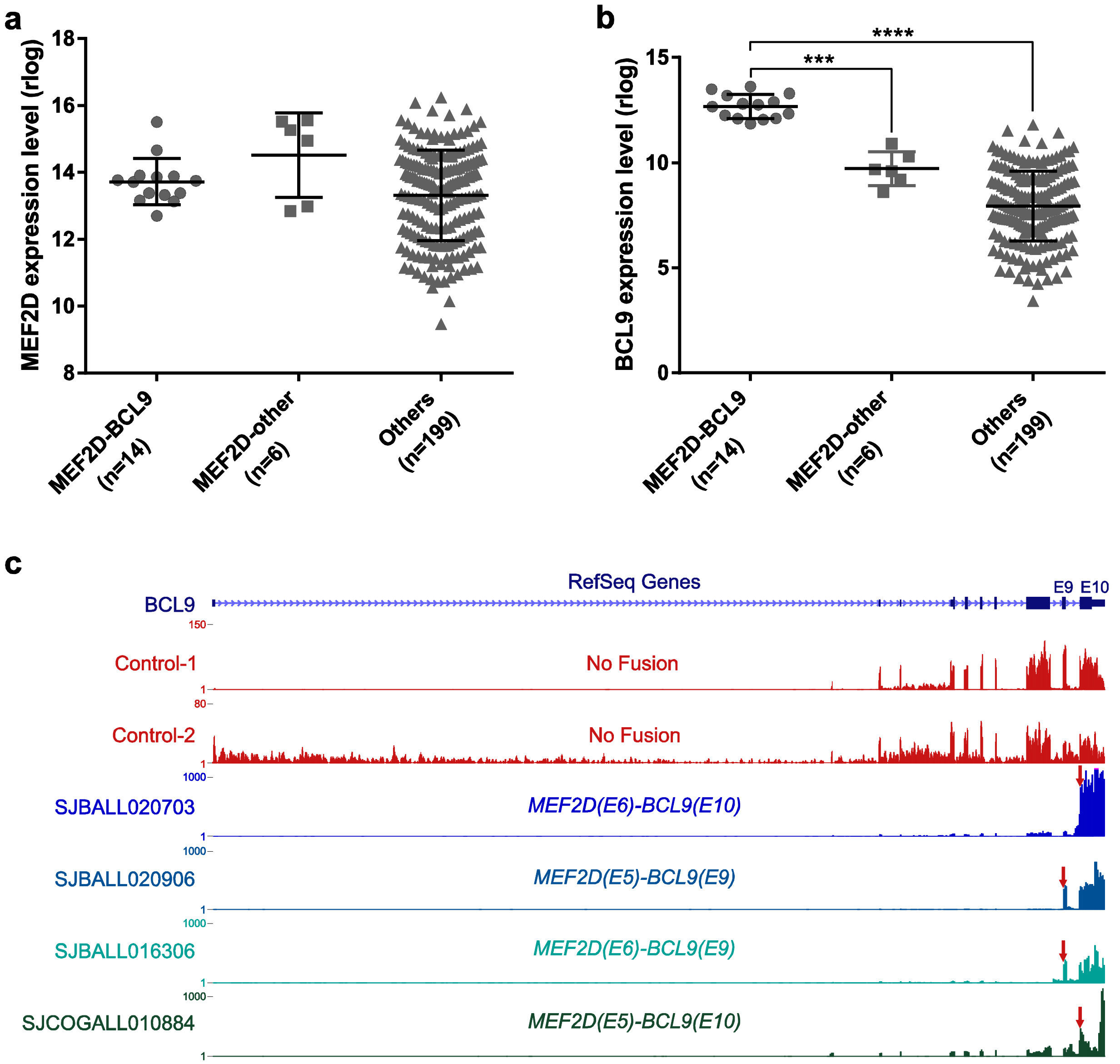

Supplementary Figure 2. Gene expression of *MEF2D* and *BCL9* according to *MEF2D* rearrangement status.

The samples used for gene expression analysis are divided into 3 groups: (1) samples with *MEF2D-BCL9* fusion, (2) other *MEF2D* fusions (MEF2D-others) (3) or without *MEF2D* rearrangement (Others). The gene expression levels for *MEF2D* (**a**) and *BCL9* (**b**) are shown in rlog. One-way ANOVA test was performed and followed by Tukey's multiple comparisons test. ***, $P \leq 0.001$; ****, $P \leq 0.0001$. **c**, Bigwig files are displayed in UCSC Genome Browser with 2 B-ALL samples without *MEF2D* rearrangement and 4 samples with different *MEF2D-BCL9* fusion isoforms showing lack of *BCL9* expression in non-rearranged B-ALL samples, and elevated expression distal to the *BCL9* rearrangement break points. RNAseq library for "Control-1" is unstranded polyA-selected mRNA library while "Control-2" is stranded total RNA library. Translocation break points from exon 9 (E9) or 10 (E10) for each *MEF2D-BCL9* fusion isoforms are indicated by red arrows.

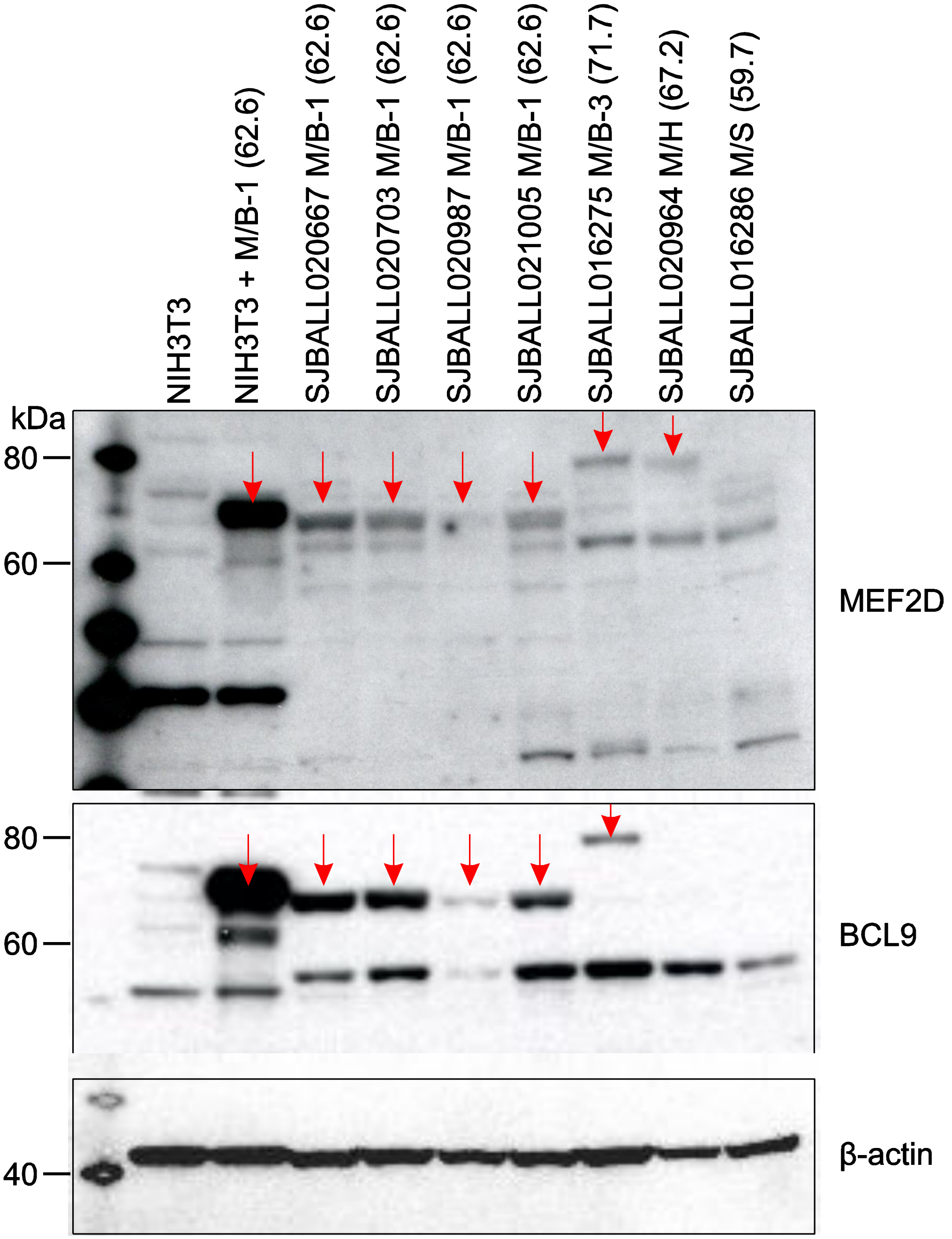

Supplementary Figure 3. Identification of MEF2D fusion protein expression by immunoblotting.

M/B-1 and M/B-3, two MEF2D-BCL9 isoforms; M/H, MEF2D-HNRNPUL1; M/S, MEF2D-SS18. Molecular weight of markers is indicated on the left side in kilodalton (kDa). Sample names are aligned above the corresponding lanes, with expected protein weights in kDa provided in parenthesis. Primary antibodies are shown on the right side. The bands corresponding to the fusion proteins are indicated by red arrows. Samples with indistinguishable target bands are not marked.

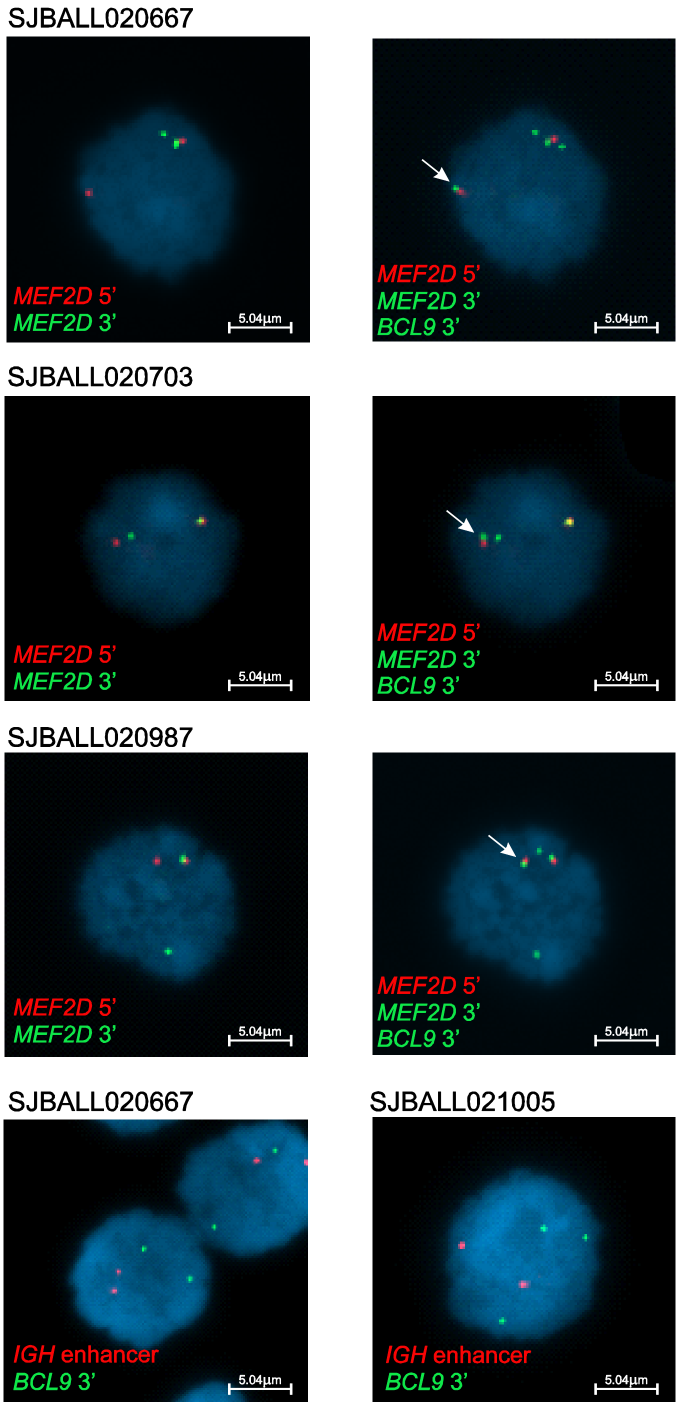

Supplementary Figure 4. Fluorescence in situ hybridization confirms *MEF2D* rearrangements.

For the top three rows of images, sequential FISH was performed first with probes for the 5' (red) and 3' (green) regions of *MEF2D* to demonstrate disruption of the locus. The second hybridization used a probe to the 3' region of *BCL9* (green, right panel) to confirm rearrangement of the two loci. White arrows in the right hand panels indicate the fusions. The images in the last row show lack of *IGH* to *BCL9* fusion.

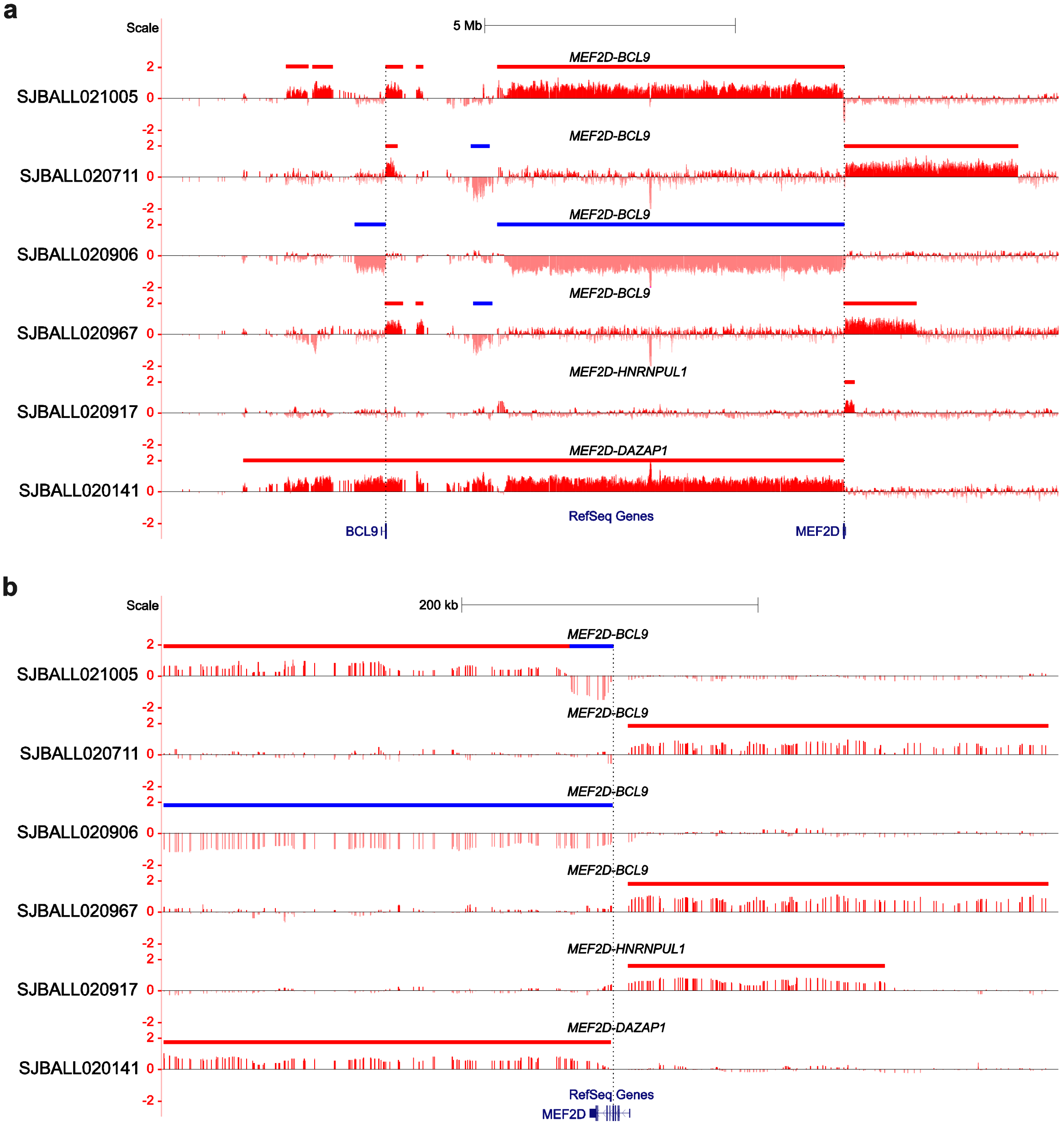

Supplementary Figure 5. DNA copy number alterations at *MEF2D* and partner loci.

Single nucleotide polymorphism (SNP) 6.0 microarray data is shown for representative samples as $\log_2$ ratio copy number data aligned to hg18 in the UCSC genome browser (track may be accessed at http://genome.ucsc.edu/cgi-bin/hgTracks?hgS_doOtherUser=submit&hgS_otherUserName=cmulligh&hgS_otherUserSessionName=MEF2D_fusions_SNP_3_18Jan16_hg18). Copy number alterations (CNA) are shown as probe level data (vertical lines) above (copy gain) or below (copy loss) the x axis. The regions of gain and loss are marked by red and blue lines respectively. The breakpoints at *BCL9* and *MEF2D* are shown across the panels by dotted vertical lines. **a**, data spanning from *BCL9* to *MEF2D* region. Samples with *MEF2D-BCL9* fusions were observed with CNA breakpoints at *BCL9*, but not for samples with other *MEF2D* rearrangements; all *MEF2D*-rearranged cases were observed with CNA breakpoints at *MEF2D*. **b**, data zoomed in for *MEF2D*.

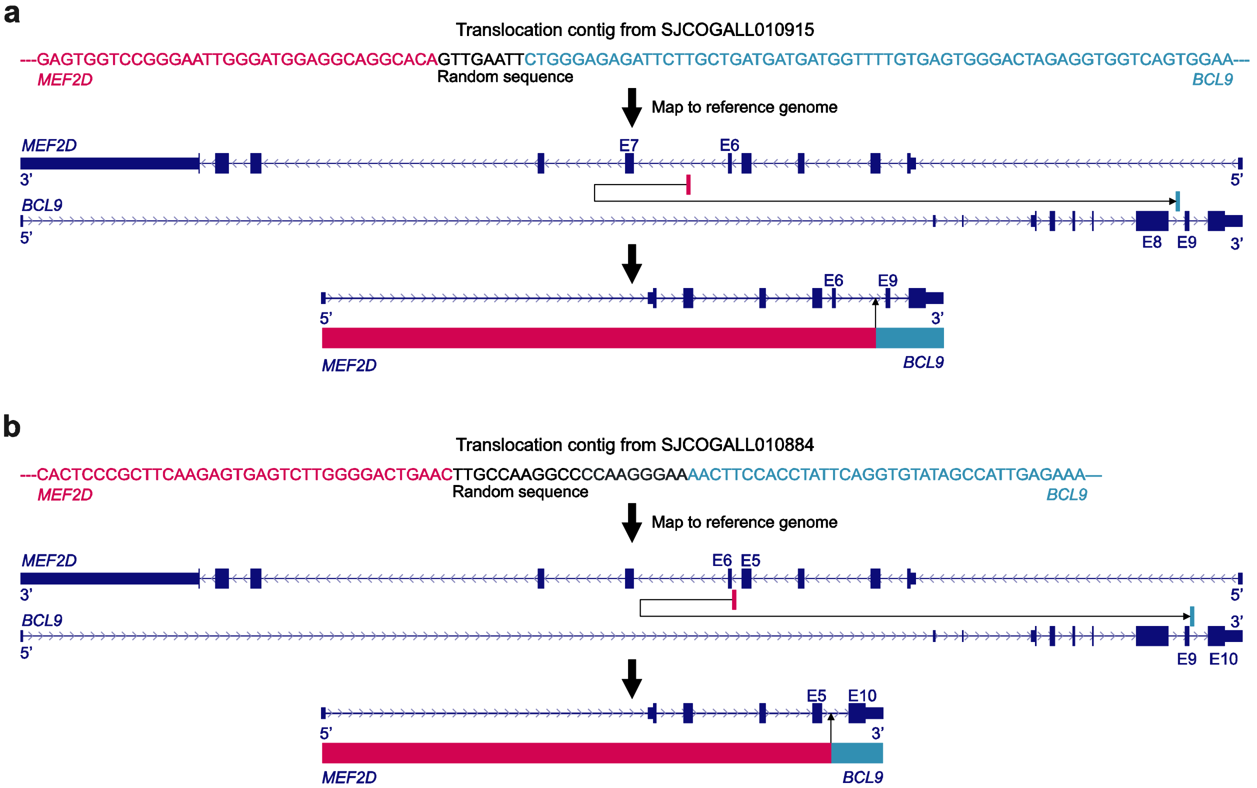

Supplementary Figure 6. Genomic rearrangements of *MEF2D-BCL9*.

Two cases with *MEF2D-BCL9* fusion have been whole-genome sequenced and analyzed by Complete Genomics (Complete Genomics, Inc., CA). **a**, Sample SJCOGALL010915 with translocation at 3' of exon 6 (E6) on *MEF2D* and 5' of exon 9 (E9) on *BCL9*, and **b**, SJCOGALL010884 with translocation at 3' of exon 5 (E5) on *MEF2D* and 5' of exon 10 (E10) on *BCL9*. Chromosomal break points of the *MEF2D-BCL9* rearrangements were identified in intron regions and consistent with the fusions identified by RNAseq data (Fig. 1). The assembled contigs are presented and the sequences from *MEF2D*, *BCL9* and random insertions are shown in red, blue and dark colors, respectively. The contigs are mapped to human reference genome and shown in UCSC genome browser, with *MEF2D* and *BCL9* segments indicated by red and blue bars.

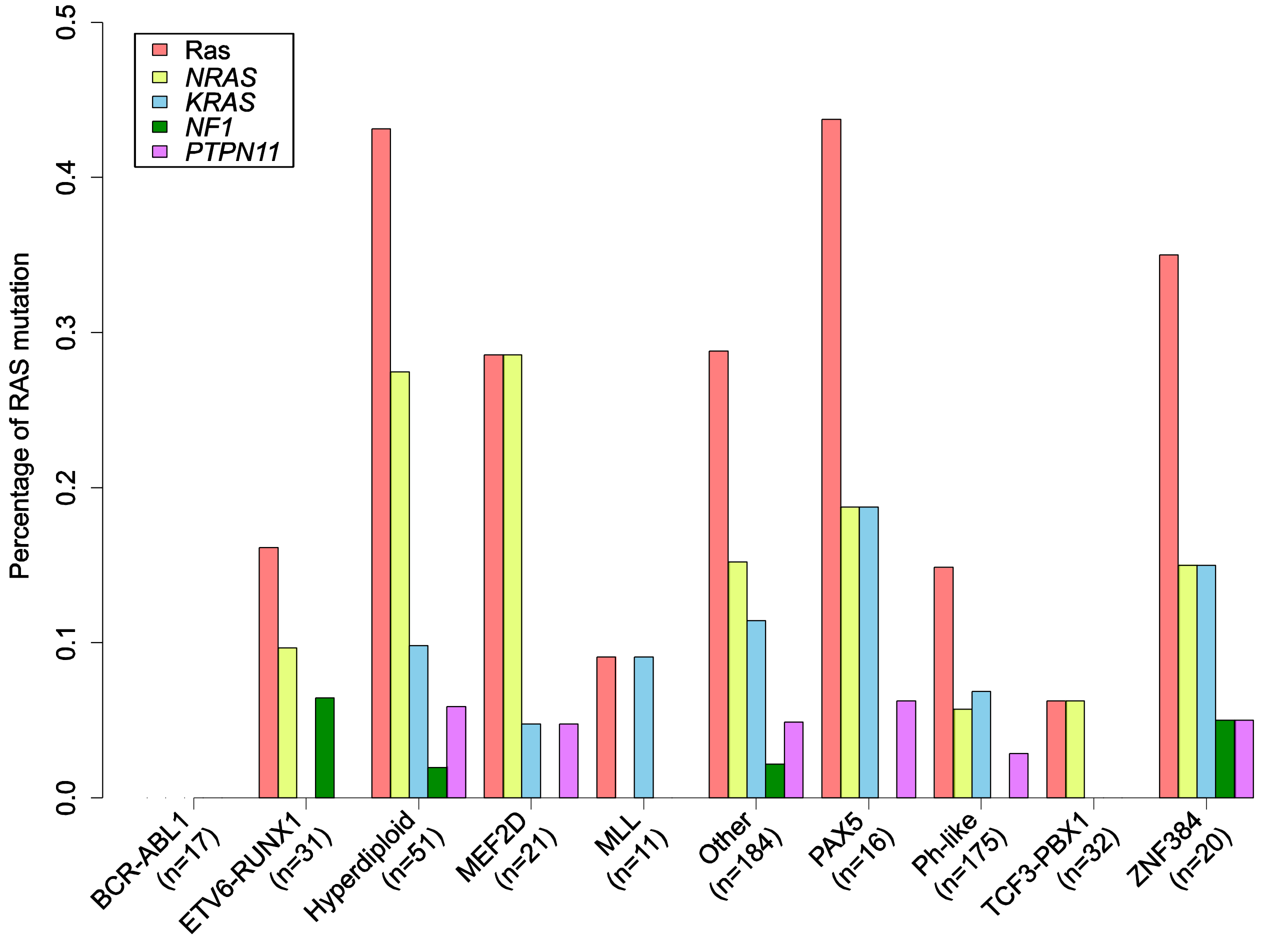

Supplementary Figure 7. Ras pathway mutations in different ALL cohorts.

Ras pathway mutations (*NRAS*, *KRAS*, *NF1* and *PTPN11*) are presented by subgroups (see "Final Group", Supplementary Data 1). Only the groups with more than 10 cases are shown. "Ras" in the legend represents the four Ras pathway genes. Distribution of Ras mutations across different ALL subgroups are significantly different (Pearson's Chi-squared test, *P* value = 8.82e-06).

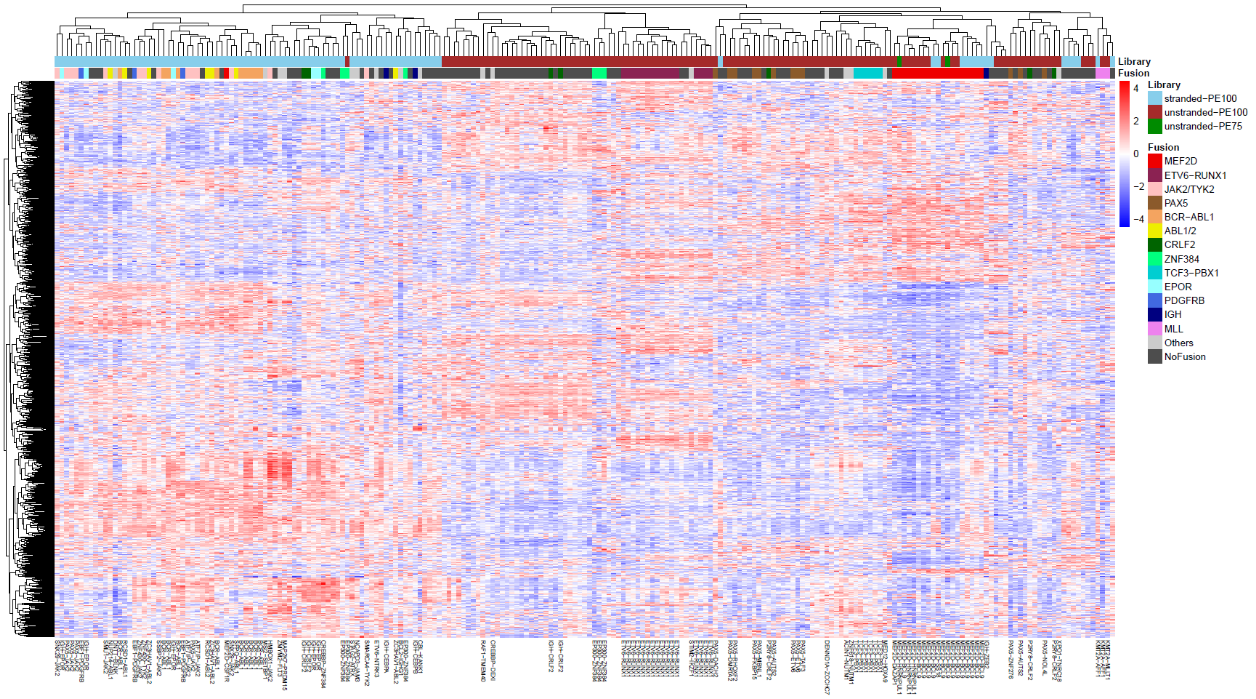

Supplementary Figure 8. Heatmap of top 1000 MAD genes in RNAseq samples.

Unsupervised hierarchical clustering of 1000 genes ranked highest median absolute variation (MAD) across the 219 samples showed clustering of 19 *MEF2D*-rearranged cases, with the exception of the case harboring *MEF2D-CSF1R*, which clustered with Ph-like ALL samples. Genes with top 800 and 500 MAD value were also tested by unsupervised clustering and also observed with similar clustering patterns, and the 19 *MEF2D*-rearranged samples were always clustered as a tight subgroup (data not shown). D30C, Depth ≥30x coverage; stranded-PE100, total stranded paired-end 100bp RNAseq; unstranded-PE100, unstranded mRNA paired-end 100bp RNAseq; unstranded-PE75, unstranded mRNA paired end 75bp RNAseq.

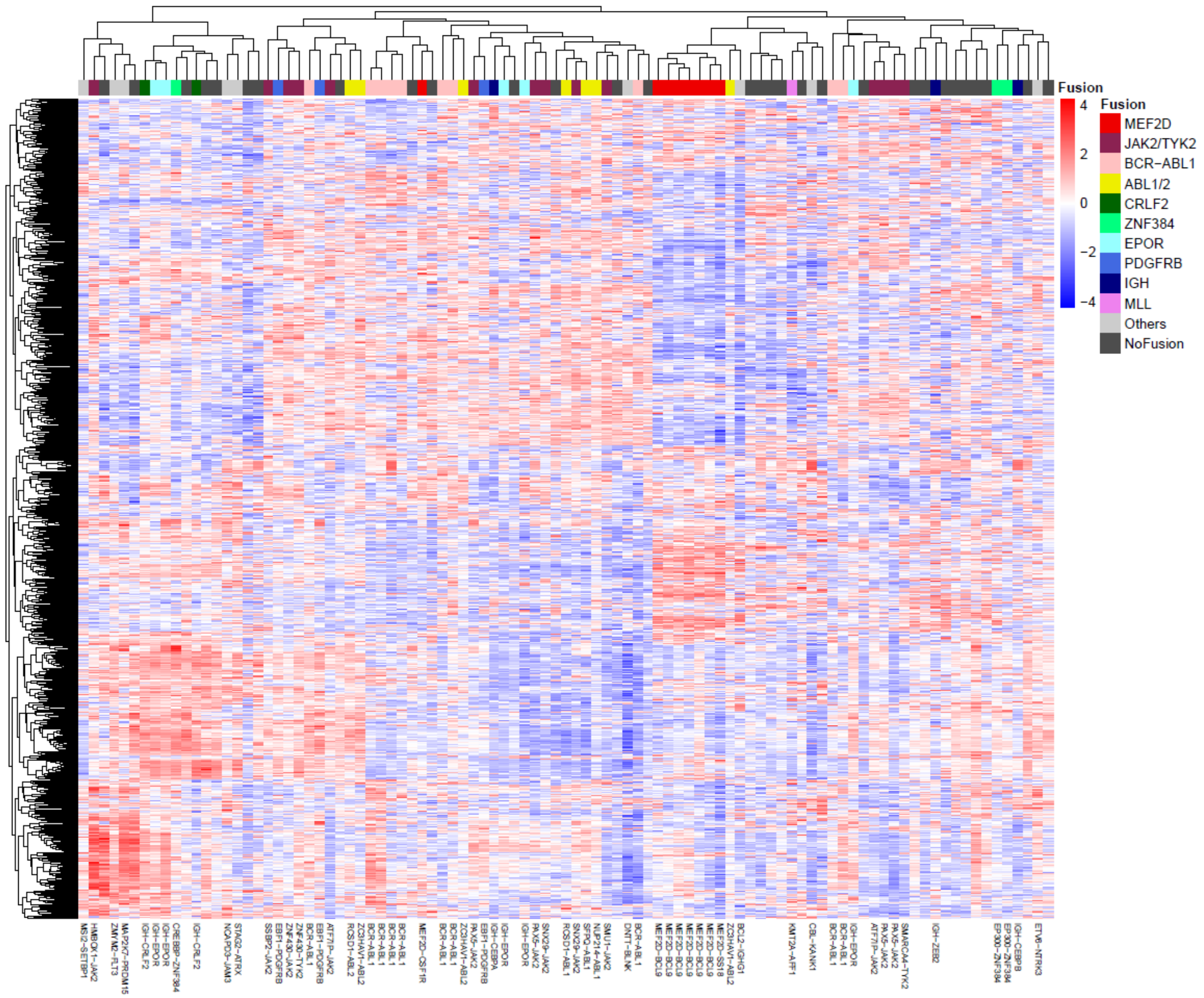

Supplementary Figure 9. Heatmap of top 1000 MAD genes in stranded RNAseq samples.

The 95 stranded samples included 8 with *MEF2D* fusions, and one is *MEF2D-CSF1R* fusion. Using the top 1000 MAD gene clustering, the *MEF2D*-rearranged cases were clustered tightly, except the one with *MEF2D-CSF1R*.

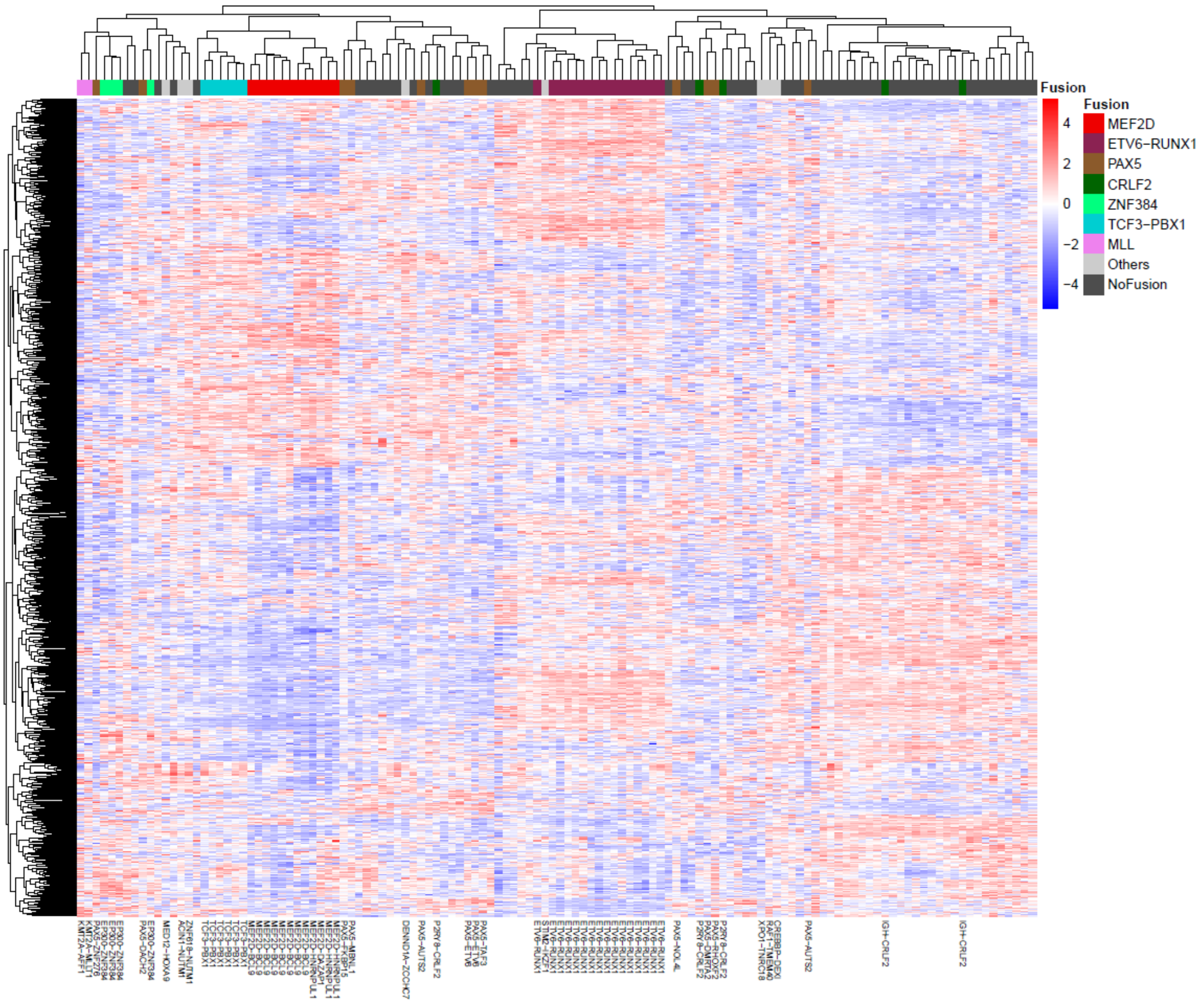

Supplementary Figure 10. Heatmap of top 1000 MAD genes in unstranded RNAseq samples.

Hierarchical clustering was performed for unstranded RNAseq data, including 12 samples with *MEF2D* rearrangements and 112 control cases.

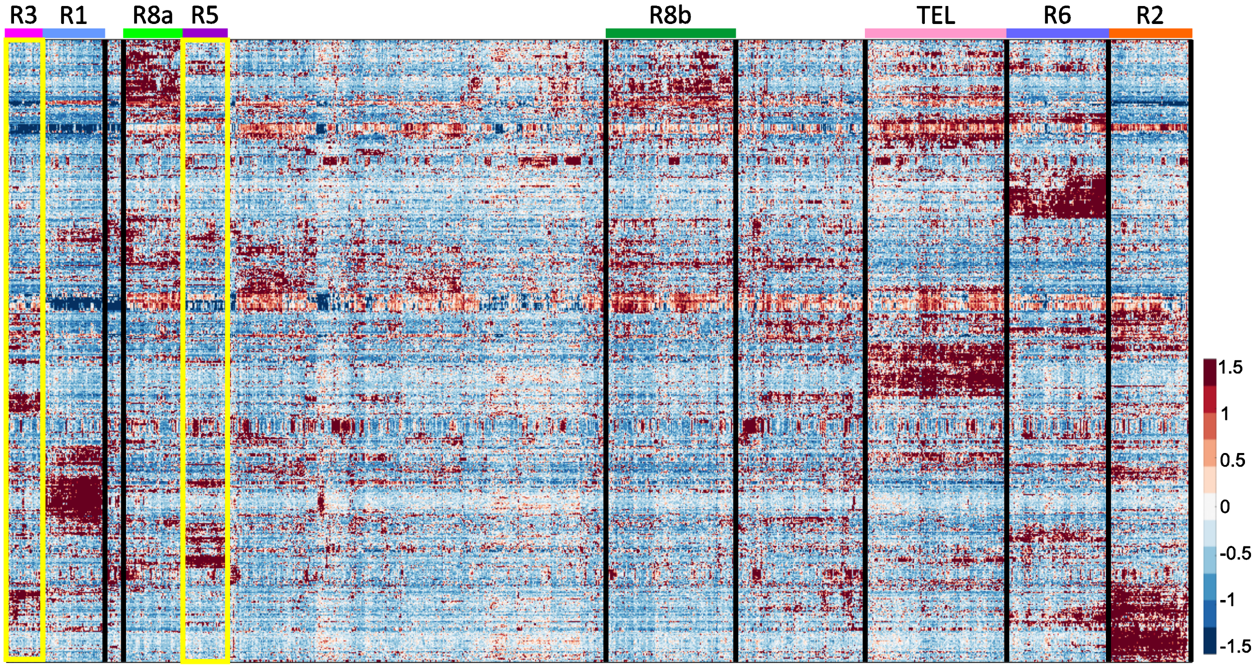

Supplementary Figure 11. R3 subgroup and *MEF2D* rearrangements.

ROSE (Recognition of Outliers by Sampling Ends) analysis[1] of 1164 cases with Affymetrix U133 Plus 2 microarray data showing clustering of R3 (the most left yellow box). This cluster includes 31 cases and 30 have been validated with *MEF2D* rearrangements. Besides, the R5 group (*ZNF384*-rearranged cases) is also marked by yellow box.

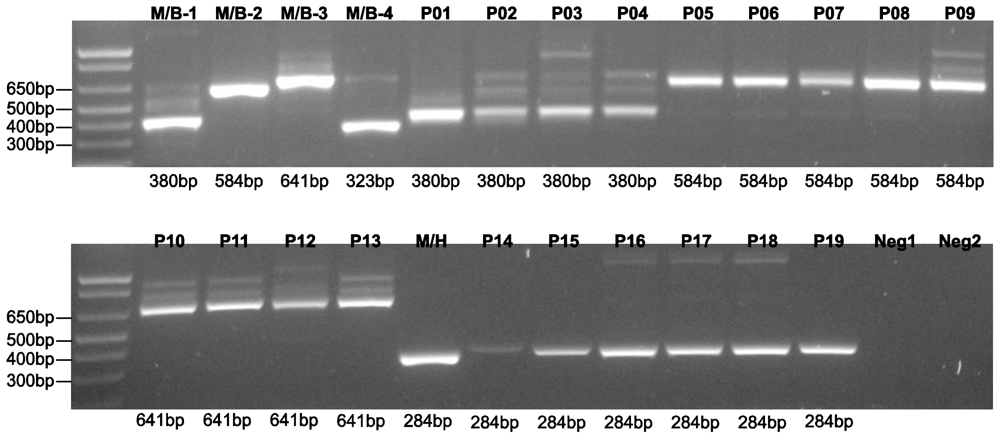

Supplementary Figure 12. ***MEF2D*** **rearrangements validation in R3 subgroup.**

Detailed information for each sample was listed in Supplementary Data 6.

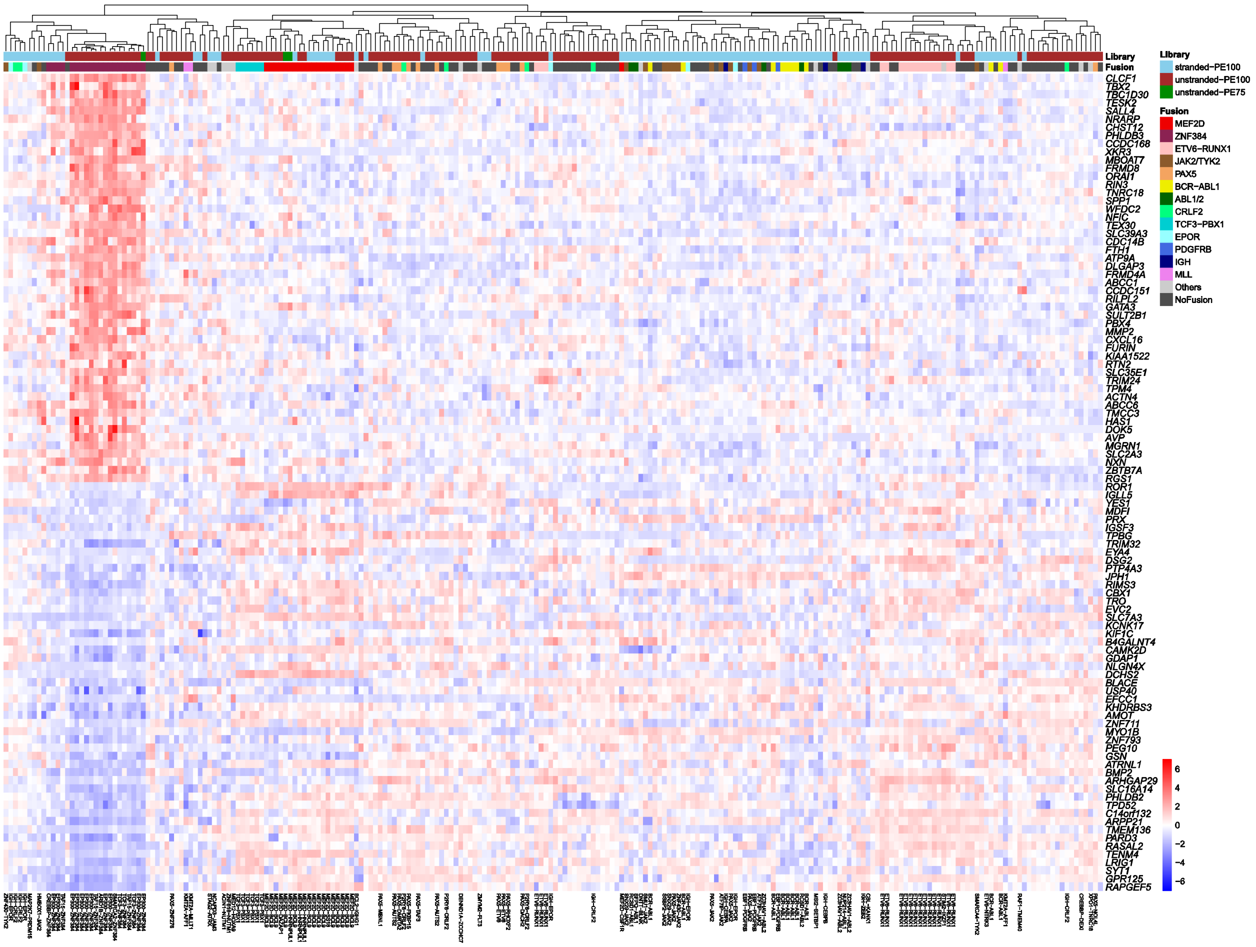

Supplementary Figure 13. Gene expression profile of *ZNF384*-rearranged ALL.

Top 50 up-regulated and top 50 down-regulated genes were selected for supervised clustering. Detailed signature gene list is provided in Supplementary Data 7.

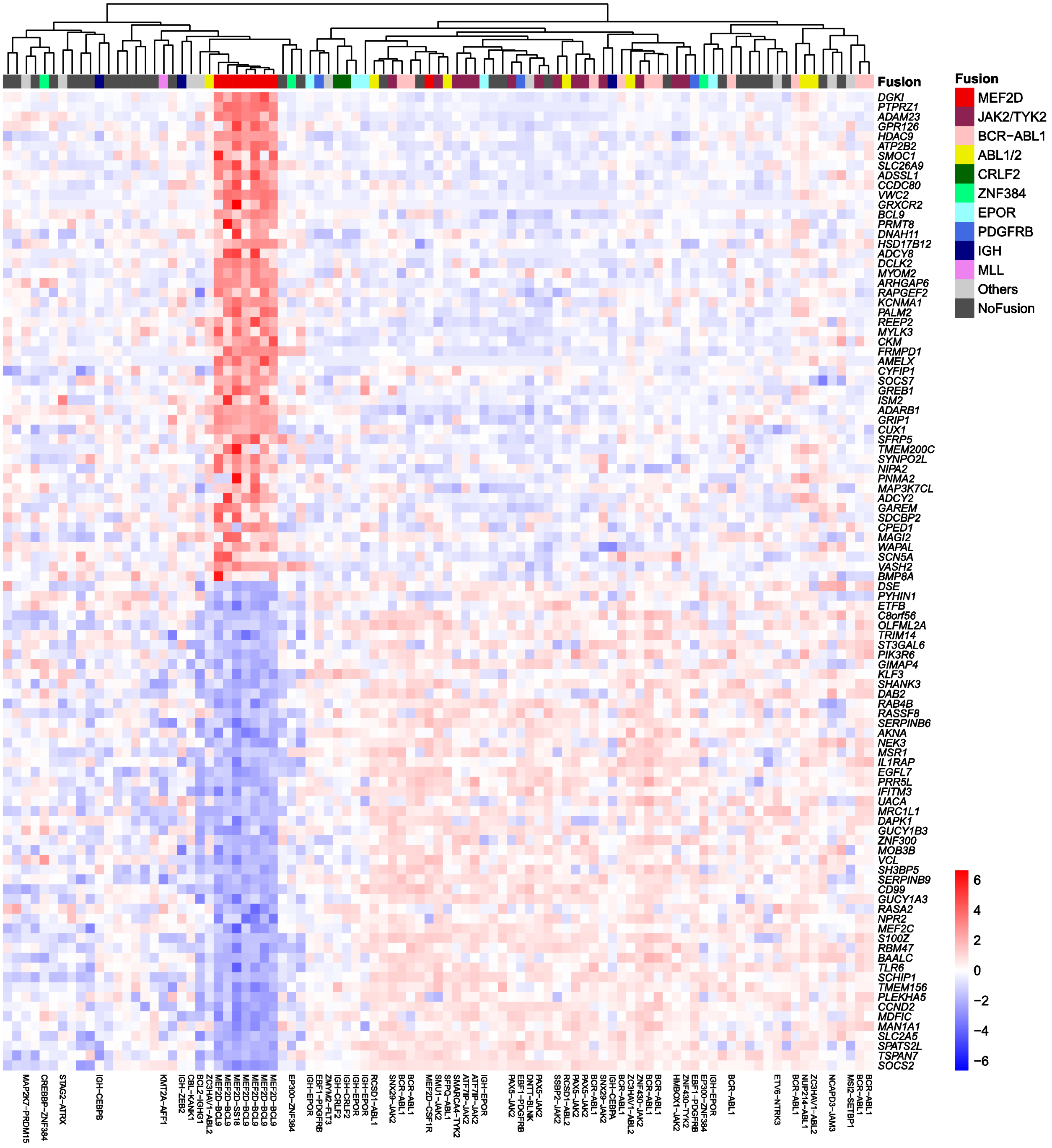

Supplementary Figure 14. Heatmap of 100 signature genes in stranded RNAseq samples.

Top 50 up-regulated and top 50 down-regulated genes were selected for supervised clustering. Detailed signature gene list is provided in Supplementary Data 4.

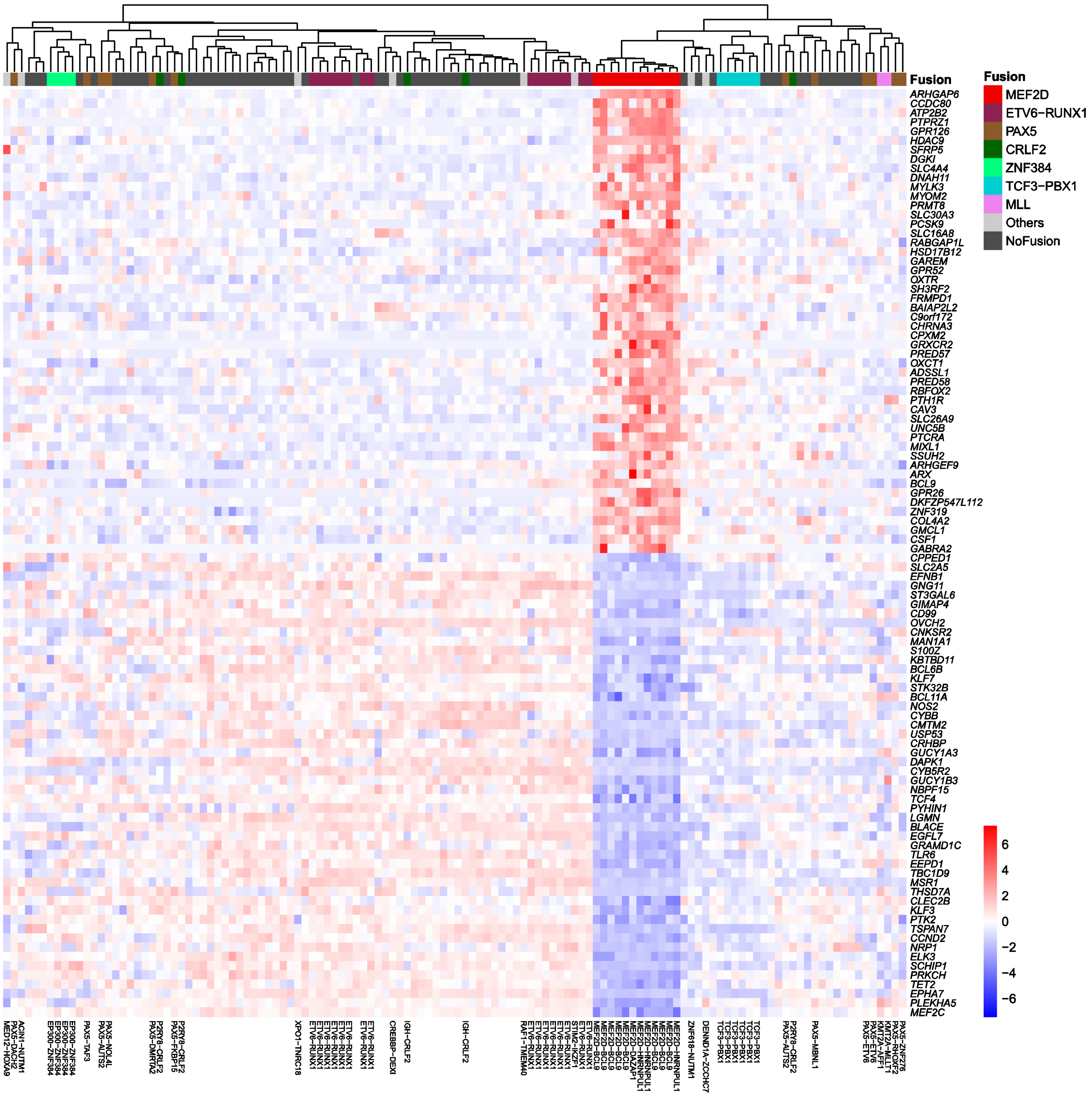

Supplementary Figure 15. Heatmap of 100 signature genes in unstranded RNAseq samples.

Top 50 up-regulated and top 50 down-regulated genes were selected for supervised clustering. Detailed signature gene list is provided in Supplementary Data 5.

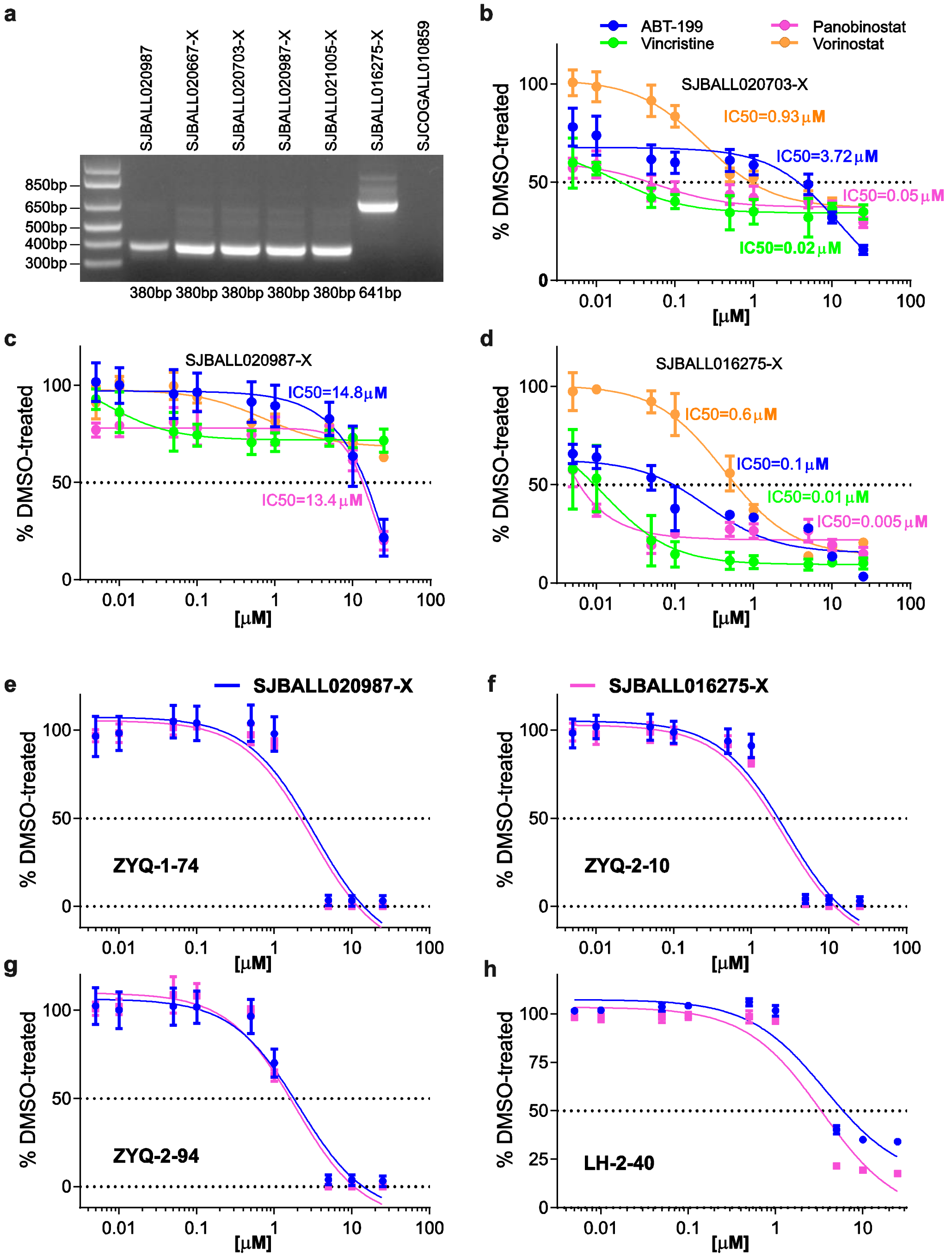

Supplementary Figure 16. Drug sensitivity assay in xenografts of ALL with *MEF2D* rearrangements.

a, Sample SJBALL020987 and SJCOGALL010859 with M/B-1 and M/H fusions are used as positive and negative control, respectively. Sample ID with "-X" suffix means the sample xenografted to mouse and harvest while the mouse developed leukemia. **b-d**, Ex vivo drug sensitive assay in 3 xenografted samples with same 4 drugs shown in Fig. 4c. **c-h**, Four BCL9 inhibitors (ZYQ-1-74, ZYQ-2-10, ZYQ-2-94 and LH-2-40) were tested in xenografted leukemia samples derived from two patients, showing IC50 values of greater than 1μM, suggestive of non-BCL9-specific killing.

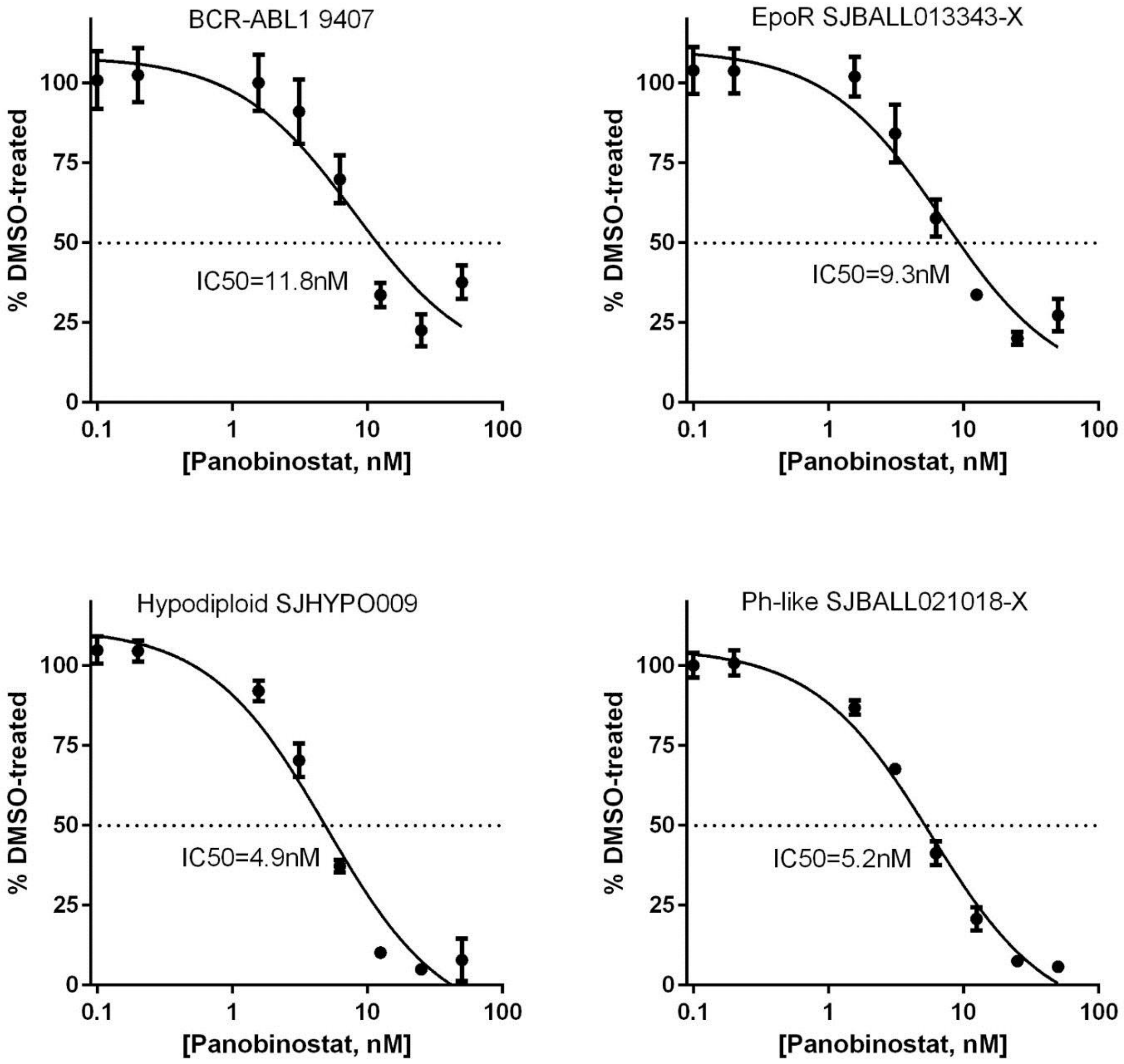

Supplementary Figure 17. Drug sensitivity of non-MEF2D-rearranged ALL to panobinostat.

Ex vivo panobinostat sensitivity test in different subtypes of B-ALL.

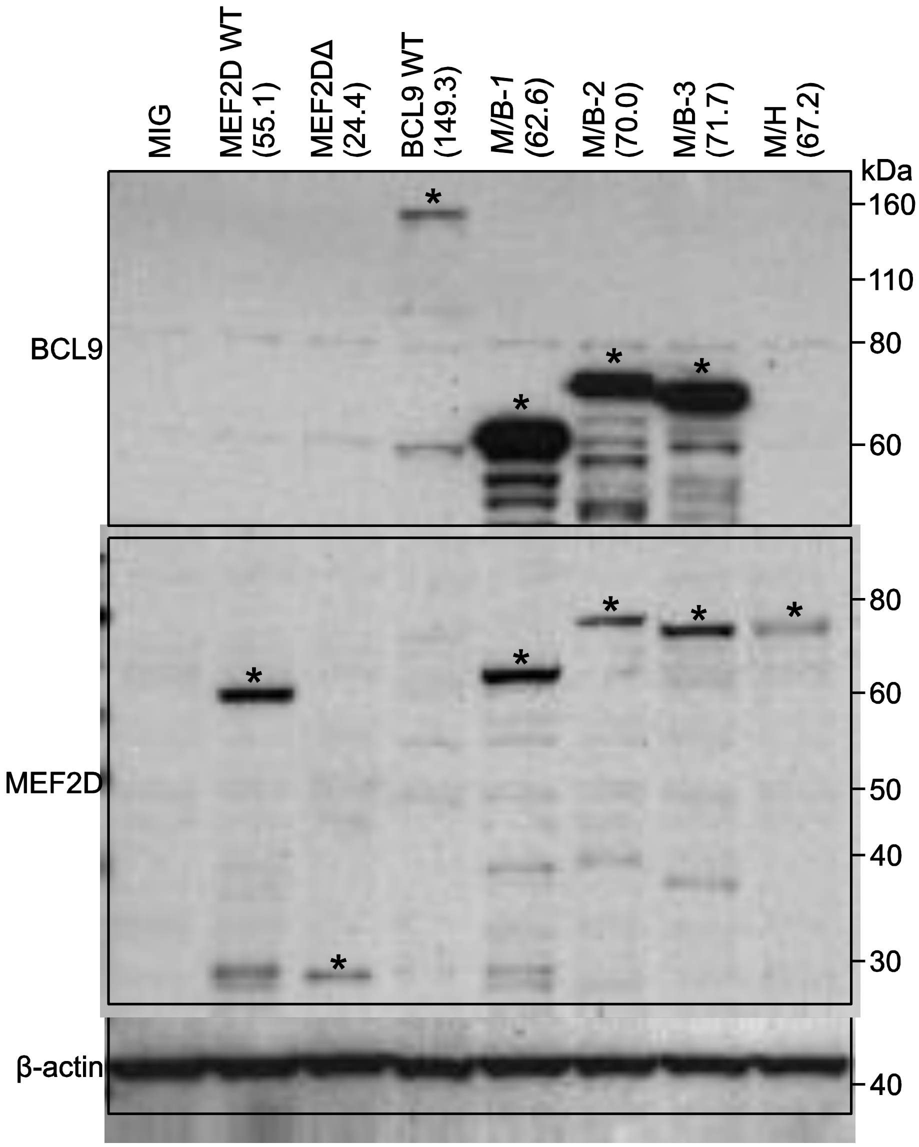

Supplementary Figure 18. Expression of proteins by retroviruses by immunoblotting.

Protein weight (kDa) is put in parenthesis at the top of each lane except MIG. Target protein is marked by “*” on top of specific band.

Supplementary Table 1. Sequencing metrics for *MEF2D*-rearranged cases

Sample ID	Duplication Rate	Average Depth	Coverage						
			>=1X	>=5X	>=10X	>=15X	>=20X	>=25X	>=30X
SJBALL013388	49.4%	33.2	66.1%	52.5%	44.8%	39.0%	34.2%	30.1%	26.7%
SJBALL014169	26.8%	67.0	69.1%	55.0%	48.8%	44.6%	41.0%	38.0%	35.4%
SJBALL015117	26.5%	53.8	66.4%	54.2%	47.4%	42.6%	38.6%	35.2%	32.2%
SJBALL016252	50.2%	55.6	67.3%	55.6%	48.7%	43.5%	39.3%	35.7%	32.5%
SJBALL016275	39.1%	70.0	68.5%	58.0%	53.1%	49.5%	46.4%	43.6%	41.1%
SJBALL016286	51.7%	22.6	63.0%	48.6%	39.9%	33.5%	28.5%	24.5%	21.2%
SJBALL016291	42.8%	37.8	67.9%	55.8%	48.8%	43.6%	39.1%	35.2%	31.8%
SJBALL016306	23.1%	55.5	68.4%	55.5%	48.5%	43.7%	39.8%	36.6%	33.7%
SJBALL020141	9.7%	99.1	69.3%	59.3%	54.2%	50.5%	47.5%	44.8%	42.3%
SJBALL020667	33.1%	77.5	64.9%	55.0%	49.7%	45.5%	41.9%	38.7%	35.9%
SJBALL020703	26.2%	83.1	68.6%	58.5%	53.4%	49.7%	46.4%	43.5%	40.8%
SJBALL020711	32.8%	94.9	67.5%	58.3%	53.3%	49.6%	46.4%	43.6%	41.0%
SJBALL020906	15.4%	63.4	65.4%	52.9%	45.9%	41.2%	37.6%	34.6%	32.2%
SJBALL020917	11.2%	86.0	69.8%	58.4%	52.2%	47.7%	44.1%	40.9%	38.2%
SJBALL020964	7.8%	93.5	68.5%	58.6%	53.9%	50.6%	47.7%	45.1%	42.7%
SJBALL020967	8.4%	96.0	65.6%	55.4%	50.6%	47.4%	44.6%	42.2%	40.0%
SJBALL020987	10.8%	106.3	67.5%	57.4%	52.6%	49.4%	46.7%	44.3%	42.1%
SJBALL021005	15.2%	129.2	72.6%	62.5%	58.0%	54.9%	52.5%	50.3%	48.4%
SJCOGALL010235	48.7%	72.1	63.5%	51.5%	44.8%	40.0%	36.2%	33.0%	30.4%
SJCOGALL010859	78.0%	43.0	64.4%	48.4%	39.6%	33.7%	29.3%	25.9%	23.1%
SJCOGALL010884*	57.5%	51.7	58.4%	41.7%	33.3%	28.2%	24.6%	22.0%	19.8%
SJCOGALL010915*	88.3%	25.0	49.7%	27.5%	19.0%	14.6%	11.9%	10.0%	8.6%

Note: Calculated based on 33.7Mb coding region according to UCSC HG19 RefSeq dataset

*RNAseq data is not enough for gene expression profiling

Supplementary Table 2. Primers used for validation of *MEF2D* fusions

Fusion ID	Gene A	Transcript A	Break A	Gene B	Transcript B	Break B	Primer F Seq	Primer R Seq
F1	*MEF2D*	NM_001271629	E6(3')	*BCL9*	NM_004326	E10(5')	AACTTTGCCATGCCTGTCACG	ACGGCATTTGGAGAGGGCATC
F2	*MEF2D*	NM_001271629	E5(3')	*BCL9*	NM_004326	E9(5')	AACTTTGCCATGCCTGTCACG	ACGGCATTTGGAGAGGGCATC
F3	*MEF2D*	NM_001271629	E6(3')	*BCL9*	NM_004326	E9(5')	AACTTTGCCATGCCTGTCACG	ACGGCATTTGGAGAGGGCATC
F4	*MEF2D*	NM_001271629	E5(3')	*BCL9*	NM_004326	E10(5')	AACTTTGCCATGCCTGTCACG	ACGGCATTTGGAGAGGGCATC
F5	*MEF2D*	NM_001271629	E8(3')	*HNRNPUL1*	NM_007040	E12(5')	TCACTTGAACAATGCCCAGCG	GGTTGTCAAAGCGCTTTTCAGG
F6	*MEF2D*	NM_001271629	E8(3')	*SS18*	NM_001007559	E6(5')	TCACTTGAACAATGCCCAGCG	GGGAGGAATCTGTCTCTGACCC
F7	*MEF2D*	NM_001271629	E6(3')	*DAZAP1*	NM_018959	E7(5')	CTCCCTGGTCACCCCTTCCC	TCCATAGCCACCAATCGCCTG
F8	*MEF2D*	NM_001271629	E7(3')	*CSF1R*	NM_001288705	E11(5')	GGTCATCCCTGCCAAGTCTCC	CTCCAGCTCCGAGGGTCTTAC
R1	*BCL9*	NM_004326	E9(3')	*MEF2D*	NM_001271629	E7(5')	CAGAGCCAACCCTTTCCCAGA	TGCATTAACCCCTTTCCTGCC
R2	*BCL9*	NM_004326	E9(3')	*MEF2D*	NM_001271629	E6(5')	CAGAGCCAACCCTTTCCCAGA	TGCATTAACCCCTTTCCTGCC
R3	*DAZAP1*	NM_018959	E6(3')	*MEF2D*	NM_001271629	E8(5')	CAGAAAGGACCCAGGAGCGAT	CAGTGACATTGCCTAGCGACA

Supplementary Table 3. Mutations identified in MEF2D-rearranged cases

Sample ID	Gene A	Break A	Gene B	Break B	Key Mutations*	SNP Results
SJBALL013388	*MEF2D*	E6(3')	*BCL9*	E10(5')		NA
SJBALL016252	*MEF2D*	E6(3')	*BCL9*	E10(5')		NA
SJBALL016291	*MEF2D*	E6(3')	*BCL9*	E10(5')	*NRAS*(p.G12C)	NA
SJBALL020667	*MEF2D*	E6(3')	*BCL9*	E10(5')	*NRAS*(p.G12D)	No *MEF2D* change
SJBALL020703	*MEF2D*	E6(3')	*BCL9*	E10(5')		No *MEF2D* change
SJBALL020987	*MEF2D*	E6(3')	*BCL9*	E10(5')		No *MEF2*D change
SJBALL021005	*MEF2D*	E6(3')	*BCL9*	E10(5')		Deletion at *MEF2D*,<br>and adjacent gain to *BCL9*
SJCOGALL010235	*MEF2D*	E6(3')	*BCL9*	E10(5')	*NRAS*(p.G12A);*KRAS*(p.A146V);<br>*PTPN11*(p.E76A)	NA
SJBALL014169	*MEF2D*	E5(3')	*BCL9*	E9(5')		NA
SJBALL020711	*MEF2D*	E5(3')	*BCL9*	E9(5')	*NRAS*(p.G12D)	Focal gains at *MEF2D* and *BCL9*
SJBALL020906	*MEF2D*	E5(3')	*BCL9*	E9(5')		Deletion between *BCL9* and *MEF2D*
SJBALL020967	*MEF2D*	E5(3')	*BCL9*	E9(5')	*NRAS*(p.G13D)	Focal gains at *MEF2D* and *BCL9*
SJBALL016275	*MEF2D*	E6(3')	*BCL9*	E9(5')	*CDKN2A*(p.W110*)	NA
SJBALL016306	*MEF2D*	E6(3')	*BCL9*	E9(5')		NA
SJCOGALL010915	*MEF2D*	E6(3')	*BCL9*	E9(5')	*IKZF1* e4-8 del	NA
SJCOGALL010884	*MEF2D*	E5(3')	*BCL9*	E10(5')	*NRAS*(p.Q61K); *IKZF1* e4-8 del	NA
SJBALL020917	*MEF2D*	E8(3')	*HNRNPUL1*	E12(5')		Focal gains at *MEF2D* and *HNRNPUL1*
SJBALL020964	*MEF2D*	E8(3')	*HNRNPUL1*	E12(5')		Focal gain at *HNRNPUL1*
SJCOGALL010859	*MEF2D*	E8(3')	*HNRNPUL1*	E12(5')		NA
SJBALL016286	*MEF2D*	E8(3')	*SS18*	E6(5')		NA
SJBALL020141	*MEF2D*	E6(3')	*DAZAP1*	E7(5')		Gain from *MEF2D* to 1cent;<br>Gain from 19p to *DAZAP1*
SJBALL015117	*MEF2D*	E7(3')	*CSF1R*	E11(5')	*IKZF1* e4-7 del (IK6)	NA

*Detected from RNAseq data

Supplementary Table 4. Immunophenotype of MEF2D-rearranged cases

Sample ID	USI	Protocol	immunophenotype	CD19	CD10	CD20	CD38	CD45	CD58	CD34	CD9	Myeloid(13/33)
SJBALL020667	PAPAEB	AALL0232	B-Precursor	P	3	N	H	P	P	N	P	N
SJBALL020703	PAPHHF	AALL0232	B-Precursor	P	2-3	N	H	P	P	N	P	N
SJBALL020711	PAPIHY	AALL0232	B-Precursor	P	2-3	Subset	H	P	P	N	P	N
SJBALL020964	PARAMR	AALL0232	B-Precursor	P	2-3	N	H	P	P	N	P	N
SJBALL020967	PAREPX	AALL0232	B-Precursor	P	2-3	N	H	L	P	N	P	N
SJBALL020987	PARMGY	AALL0232	B-Precursor	P	2	N	H	P	P	N	P	N
SJCOGALL010235	PARPNM	AALL0232	B-Precursor	P	1-2	Variable	H	P	P	N	P	N
SJBALL021005	PARTGX	AALL0232	B-Precursor	P	2	N	H	L	P	Subset	P	N
SJCOGALL010915	PASUBW	AALL0232	B-Precursor	P	N	N	H	N	P	Subset	P	N
SJCOGALL010884	PATTEE	AALL0232	B-Precursor	P	2	Positive	H	P	P	N	P	Positive
SJBALL016306	PAUZLX	AALL1131	B-Precursor	P	2	N	H	P	P	Subset	P	Subset
SJBALL016252	PAVFLB	AALL1131	B-Precursor	P	N	N	Positive	P	L	N	P	N
SJBALL013388	PAVRYA	AALL1131	B-Precursor	P	N	N	H	P	P	Subset	P	N
SJBALL016291	PAWAYL	AALL1131	B-Precursor	P	4	Variable	H	P	P	P	Part	N

P=positive at an intensity typical for most cases of ALL; this is usually higher than normal cells for CD58 and CD9, and comparable to normal for CD19 and CD45

N=Negative; includes cases with only focal (<10%) positive cells

H=High; at the level of normal hematogones

L=Low

For CD10, the number refers to the log decade of positivity. Most cases of B ALL would score as 3 or 3-4

Supplementary Table 5. Primers used for cloning and validate retroviral constructs

Target	Original sample	Forward primer-clone	Reverse primer-clone	Forward primer-validation	Reverse primer-validation
MEF2D-WT	SJBALL020967	CTCCGTAGCACTGAGGACCC	TGAGAGGAGGGGAGTGGGAAT	CCTAAGCCTCCGCCTCCTCTTCCTC;<br>AACTTTGCCATGCCTGTCACG	AAACGCACACCGGCCTTATTCCAAG
Truncated *MEF2D**	SJBALL013388	GGGGACAAGTTTGTACAAAAAAGCAG-<br>GCTATGGGGAGGAAAAAGATTCAGATC	GGGGACCACTTTGTACAAGAAAGC-<br>TGGGTTCAAACAGGGCTGGGGCA	CCTAAGCCTCCGCCTCCTCTTCCTC	AAACGCACACCGGCCTTATTCCAAG
BCL9-WT	MOLT4<br>cell line	TCAATCAATGCATTCCAGTAACCCT	GCACATCCCATCTTAGCAGCTTAA	CCTAAGCCTCCGCCTCCTCTTCCTC;<br>CCTCAACACAGCTCCTCCAGTT;<br>ACCCAGCCACTTTCTCACTCC;<br>CTGCAGCCAACTCCACCCATTC;<br>TCCCTGGATTTGCAGGCATGAT	AAACGCACACCGGCCTTATTCCAAG;<br>ACGGCATTTGGAGAGGGCATC;<br>GAGTGGAGCTGGCAGGACTTC;<br>GTCTAGATGCAGGGTTGGGGAC;<br>CAGAGGGTTAATGCCTGGGGAA
MEF2D-BCL9-1	SJBALL013388	CTCCCCTCCGTAGCACTGAG	AACTCCCTCAAAGCAGCTCTC	CCTAAGCCTCCGCCTCCTCTTCCTC;<br>AACTTTGCCATGCCTGTCACG;<br>ACCCAGCCACTTTCTCACTCC	AAACGCACACCGGCCTTATTCCAAG
MEF2D-BCL9-2	SJBALL016306	CTCCCCTCCGTAGCACTGAG	AACTCCCTCAAAGCAGCTCTC	CCTAAGCCTCCGCCTCCTCTTCCTC;<br>AACTTTGCCATGCCTGTCACG;<br>ACCCAGCCACTTTCTCACTCC	AAACGCACACCGGCCTTATTCCAAG
MEF2D-BCL9-3	SJBALL020967	CTCCCCTCCGTAGCACTGAG	AACTCCCTCAAAGCAGCTCTC	CCTAAGCCTCCGCCTCCTCTTCCTC;<br>AACTTTGCCATGCCTGTCACG;<br>ACCCAGCCACTTTCTCACTCC	AAACGCACACCGGCCTTATTCCAAG
MEF2D-HNRNPUL1	SJCOGALL010859	CCGGAGAAGATGGGGAGGAAAA	ACTGGCTACTGTGTACTTGTGCC	CCTAAGCCTCCGCCTCCTCTTCCTC;<br>TCACTTGAACAATGCCCAGCG	AAACGCACACCGGCCTTATTCCAAG;<br>GGTTGTCAAAGCGCTTTTCAGG

* Cloned by Gateway protocol

SUPPLEMENTARY REFERENCES

1. Harvey RC, *et al.* Identification of novel cluster groups in pediatric high-risk B-precursor acute lymphoblastic leukemia with gene expression profiling: correlation with genome-wide DNA copy number alterations, clinical characteristics, and outcome. *Blood* **116**, 4874-4884 (2010).
